# CD36-mediated metabolic crosstalk between tumor cells and macrophages affects liver metastasis

**DOI:** 10.1038/s41467-022-33349-y

**Published:** 2022-10-02

**Authors:** Ping Yang, Hong Qin, Yiyu Li, Anhua Xiao, Enze Zheng, Han Zeng, Chunxiao Su, Xiaoqing Luo, Qiannan Lu, Meng Liao, Lei Zhao, Li Wei, Zac Varghese, John F. Moorhead, Yaxi Chen, Xiong Z. Ruan

**Affiliations:** 1 Centre for Lipid Research & Key Laboratory of Molecular Biology for Infectious Diseases (Ministry of Education), Institute for Viral Hepatitis, Department of Infectious Diseases, the Second Affiliated Hospital, Chongqing Medical University, Chongqing, China; 2 John Moorhead Research Laboratory, Centre for Nephrology, University College London Medical School, Royal Free Campus, University College London, London, UK

**Keywords:** Cancer microenvironment, Cancer metabolism, Tumour immunology

## Abstract

Liver metastasis is highly aggressive and treatment-refractory, partly due to macrophage-mediated immune suppression. Understanding the mechanisms leading to functional reprogramming of macrophages in the tumor microenvironment (TME) will benefit cancer immunotherapy. Herein, we find that the scavenger receptor CD36 is upregulated in metastasis-associated macrophages (MAMs) and deletion of CD36 in MAMs attenuates liver metastasis in mice. MAMs contain more lipid droplets and have the unique capability in engulfing tumor cell-derived long-chain fatty acids, which are carried by extracellular vesicles. The lipid-enriched vesicles are preferentially partitioned into macrophages via CD36, that fuel macrophages and trigger their tumor-promoting activities. In patients with liver metastases, high expression of CD36 correlates with protumoral M2-type MAMs infiltration, creating a highly immunosuppressive TME. Collectively, our findings uncover a mechanism by which tumor cells metabolically interact with macrophages in TME, and suggest a therapeutic potential of targeting CD36 as immunotherapy for liver metastasis.

## Abstract (web summary)

Macrophage-mediated immune suppression contributes to poor outcome in liver metastasis. Here the authors show that CD36-expressing metastasis associated macrophages engulf tumor cell-derived extracellular vesicles enriched in long-chain fatty acids, acquiring a pro-tumorigenic phenotype in a preclinical liver metastasis model.

## Introduction

Metastasis is the leading cause of cancer-related death, and the liver is the most common site of cancer metastases. Patients with liver metastases are strongly associated with poor prognosis and diminished therapeutic responsiveness. Cancer immunotherapy harnessing the immune system to battle tumors has achieved unprecedented success in the treatment of multiple malignancies. Nevertheless, clinical benefits from immunotherapy are observed in only 15–20% of patients with liver metastases^1,2^. The massive accumulation of macrophages in liver metastasis greatly contributes to reduced response to immunotherapy and poor outcome. Targeting macrophages may improve cancer immunotherapy for treating liver metastasis^3^.

Macrophages, the most abundant myeloid cells infiltrating the tumor microenvironment (TME), are endowed with a protumoral M2-like phenotype, facilitating tumor initiation, progression and metastasis^4^. The functional reprogramming of macrophages is a complex process which is not necessarily dependent on IL-4 and has not yet been elucidated. Emerging evidence suggests that macrophages undergo metabolic changes to adapt to their local TME, with alterations of glucose, lipid, and glutamine metabolism^5^. These changes shape their tumor-promoting phenotype with immunosuppressive and anti-inflammatory activities. There has been growing interest in the possible role of lipid homeostasis in controlling the functional state of macrophages^6–10^. Intratumoral macrophages show enhanced lipid accumulation and favor the use of fatty acids to fuel mitochondrial oxidative phosphorylation. However, the TME factors that regulate the lipid reprogramming of macrophages remain elusive.

Due to high metabolic activity of tumor cells, the TME was usually characterized by hypoxia, acidity, and nutrient depletion. Besides, tumor-related metabolites, such as nitric oxide, reactive oxygen species, and adenosine influence the composition of the TME and the function of tumor-infiltrating immune cells^11^. Increasing evidence suggests that activation of lipid metabolism in tumors, providing an abundance of lipid metabolic products, leads to tumor development and local TME diversity^12^. It has been reported that the immunosuppressive phenotype of tumor-associated macrophages is controlled by long-chain fatty acids (LCFAs) metabolism, specifically oleic acid^13^. However, it is uncertain whether specific lipid metabolites derived from tumor cells may be involved in the metabolic and functional remodeling of macrophages.

In the present study, we find that some types of LCFAs released from tumor cells shape a lipid-enriched TME. Intriguingly, the LCFAs released by tumor cells are not delivered by a carrier protein, but by extracellular vesicles. In response to the TME change, the scavenge receptor CD36, a central regulator for lipid metabolism, is upregulated in tumor-infiltrating macrophages. CD36 mediates the internalization of tumor-associated lipids, which subsequently drives the metabolic and functional reprogramming of macrophages. Furthermore, in a preclinical model of liver metastasis, the absence of CD36 in macrophages restores liver CD8^+^T cell immunity and suppresses metastatic tumor growth. Overall, our findings highlight the importance of tumor-derived lipids in educating macrophages and promoting liver metastasis via a CD36-dependent mechanism.

## Results

### Host CD36 expression is essential for liver metastasis

**Fig. 1:**
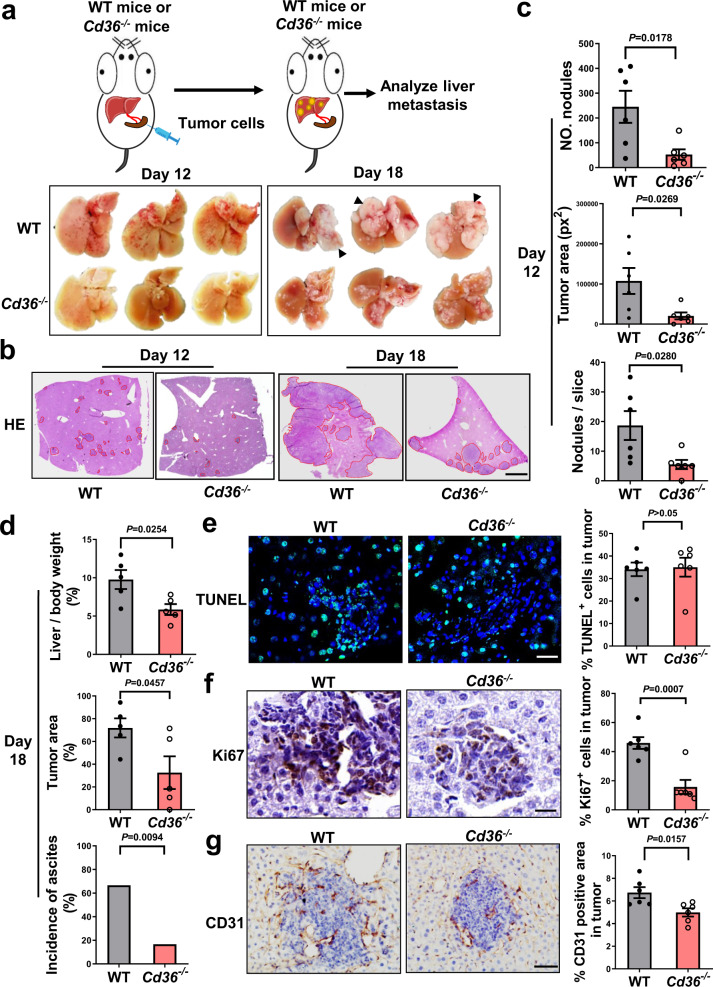
Host CD36 expression promotes tumor metastatic to the liver. 1 × 10^6^ LLC tumor cells were intrasplenically injected into WT and *Cd36*^−/−^ mice. **a** Representative metastasized livers from WT and *Cd36*^−/−^ mice on day 12 and day 18 postinjection (*n* = 6). **b** HE-stained liver sections from WT and *Cd36*^−/−^ mice on day 12 and day 18 postinjection (*n* = 6). Scale bar, 1000 μm. **c**, The number of tumor nodules (up), and the area (middle) and number of metastasized foci (down) were quantified on day 12 (*n* = 6). **d** The percentage of liver to body weight (up, *n* = 5), histopathologic tumor load (middle, *n* = 5) and the incidence of ascites (down, *n* = 6) were shown on 18 days. **e** TUNEL analysis of liver sections from WT and *Cd36*^−/−^ mice on 12 days along with the quantification (*n* = 6). Scale bar, 30 μm. **f**, **g** Immunohistochemistry staining of Ki67 (**f**, scale bar, 30 μm) and CD31 (**g**, scale bar, 100 μm) in metastasized foci from the WT and *Cd36*^−/−^ mice on 12 days along with the quantification (*n* = 6). Data are representative of two independent experiments with similar results. Values for n represent biologically independent samples. Data are mean ± SEM and P values were determined by unpaired two-tailed Student’s t-test. Source data are provided as a Source Data file.

Tumor cells with elevated CD36 expression exhibit a unique ability to initiate metastasis in a tumor cell-intrinsic manner^14^. In the present study, we evaluated whether CD36 in tumor cell-extrinsic microenvironment could influence liver metastasis. LLC cells with a high metastatic potency were intrasplenically injected into *Cd36*^−/−^ mice and their WT littermates (Fig. 1a). As shown in Fig. 1a–d, metastatic tumor formation in the liver was largely suppressed in *Cd36*^−/−^ mice compared with WT mice. To confirm the protective role of CD36 deficiency, we evaluated liver metastasis with different types of tumor cells, including the murine B16F10 melanoma cells, Hepa1-6 hepatoma cells and CT26 colon carcinoma cells. Similar to observations with the LLC cells, all these different cells showed a reduced formation of liver metastases when CD36 was absent in mice (Supplementary Fig. 1a–e).

To determine whether host CD36 affects the extravasation of tumor cells, we labeled LLC cells with CMFDA and monitored their presence in the liver. WT and *Cd36*^−/−^ mice had comparable numbers of tumor cells reached at the livers at 1 h postinjection (Supplementary Fig. 2a, b). Their arrest in the liver declined steadily after arrival, but no significant difference was observed between genotypes (Supplementary Fig. 2a, b). Hepatic expression of inflammatory factors (TNFα, IL-1β, IL-6) and endothelial adhesion molecules (E-selectin, ICAM, VCAM) were markedly induced soon after tumor cells arrived, but most of them did not differ significantly among WT and *Cd36*^−/−^ mice (Supplementary Fig. 2c, d). These data excluded a possible contribution of host CD36 to the regulation of tumor cell adhesion or extravasation. On the other hand, histological analysis revealed no significant change in tumor cell apoptosis but noted a reduction in tumor cell proliferation and angiogenesis in *Cd36*^−/−^ mice compared with WT mice (Fig. 1e–g). These data clearly show that the host expression of CD36 is critical for the colonization of tumor cells in the liver.

### CD36 expression in metastasis-associated macrophages (MAMs) contributes to liver metastasis

**Fig. 2:**
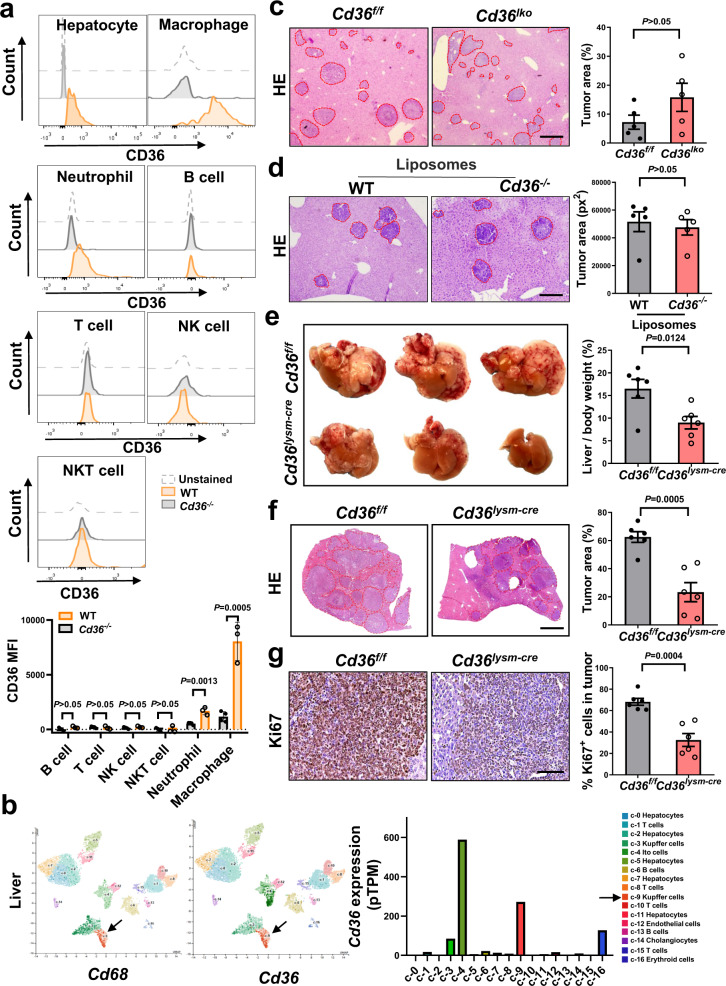
Genetic ablation of CD36 in MAMs suppresses liver metastasis. **a** The mean fluorescence intensity (MFI) of CD36 in mouse liver-resident cells analyzed by flow cytometry (*n* = 3). The corresponding *Cd36*^−/−^ cells were used as negative control. **b** CD36 expression in different cell clusters by analyzing the single-cell sequencing data of the liver from Human Protein Atlas Dataset. **c** HE staining liver sections from *Cd36*^*lko*^ mice and their littermates after intrasplenic injection of LLC cells for 14 days (*n* = 5). Quantification of tumor area was shown on the right. Scale bar, 400 μm. **d**, WT and *Cd36*^−/−^ mice were treated with clodronate liposomes twice a week after inoculation of tumor cells. On day 14, liver sections were stained with HE and the quantification of tumor area was shown on the right (*n* = 5). Scale bar, 400 μm. **e**, Representative livers from *Cd36*^*lysm-cre*^ mice and their littermates after intrasplenic injection of LLC cells (*n* = 6). The percentage of liver to body weight was shown on the right. **f**, **g** HE (**f**, scale bar, 1000 μm) and Ki67 (**g**, scale bar, 200 μm) staining in the liver sections from *Cd36*^*lysm-cre*^ mice and their littermates along with the quantification (*n* = 6). Data are representative of two independent experiments with similar results **a**, **d**, **e**, **f**. Values for *n* represent biologically independent samples. Data are mean ± SEM and *P* values were determined by unpaired two-tailed Student’s t-test. Source data are provided as a Source Data file.

We evaluated CD36 expression in distinct cell populations separated from the liver of mice without tumors using flow cytometry (Supplementary Fig. 3). In mouse liver, CD36 was expressed at the highest level in macrophages (CD45^+^CD11b^+^GR1^−^F4/80^+^), at a moderate level in hepatocytes, and at low levels in other immune cells, including neutrophils (CD45^+^CD11b^+^GR1^+^), B cells (CD45^+^CD3^−^CD19^+^), T cells (CD45^+^CD3^+^CD19^−^NK1.1^−^), NK cells (CD45^+^CD3^−^NK1.1^+^CD19^−^), NKT cells (CD45^+^CD3^+^NK1.1^+^CD19^−^) (Fig. 2a). By analyzing the single-cell sequencing data of peripheral blood mononuclear cells (PBMCs) and livers, we verified the highest level of CD36 in monocytes among PBMCs and in macrophages among liver immune cells (Fig. 2b and Supplementary Fig. 2e). Homoplastically, macrophages expressed the highest level of CD36 among splenic immune cells (Supplementary Fig. 2f). Furthermore, in metastatic liver tumors, CD36 expression was predominantly confined to MAMs (Supplementary Fig. 2g).

Previous studies suggested that hepatocyte CD36 plays a crucial role in maintaining metabolic and immune homeostasis^15^. So, we employed *Cd36*^*lko*^ mice to assess the impact of hepatocyte CD36 on the formation of liver metastasis. Metastatic tumor growth of the LLC or B16F10 cells in the liver did not differ significantly between *Cd36*^*lko*^ mice and their littermates (Fig. 2c and Supplementary Fig. 4a, b). MAMs depletion by clodronate liposomes suppressed liver metastasis (Supplementary Fig. 4c). However, the marked differences in liver metastasis between WT and *Cd36*^−/−^ mice disappeared after treated with clodronate liposomes (Fig. 2d). Then, *Cd36*^*lysm-cre*^ mice (CD36 absent in macrophages) were generated and the knockout efficiency was verified (Supplementary Fig. 4d–g). Liver metastasis was markedly restrained in the *Cd36*^*lysm-cre*^ mice versus control mice, as evidenced by reduced liver weight, decreased tumor burden and inhibited tumor cell proliferation (Fig. 2e–g and Supplementary Fig. 4h, i). Collectively, these data indicate that CD36 is effective in promoting liver metastasis in a MAM-dependent manner.

### CD36 expression is upregulated in MAMs

**Fig. 3:**
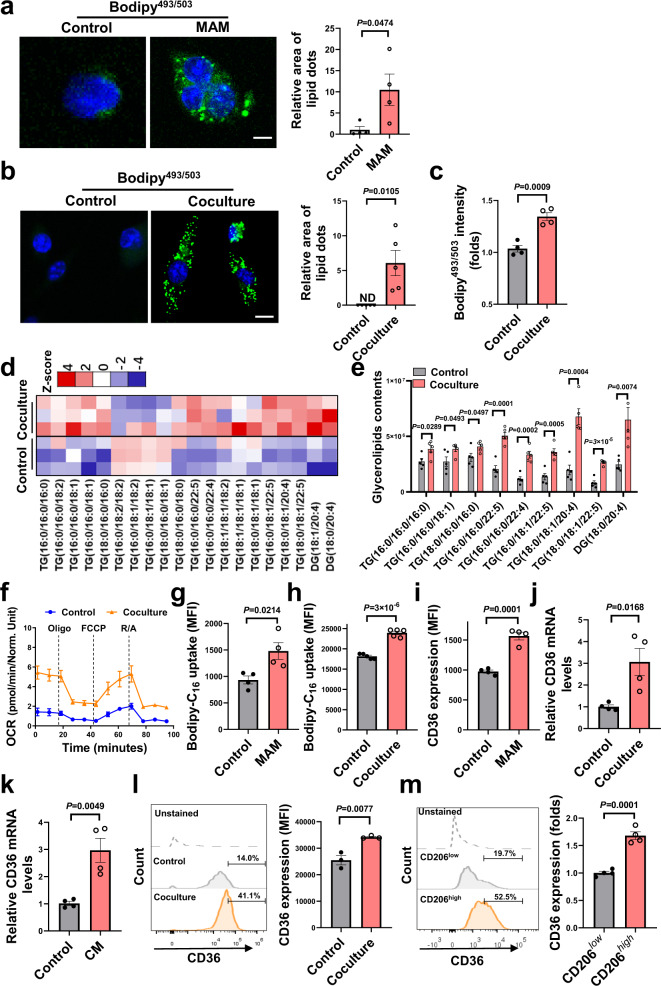
MAMs exhibit increased lipid deposition and higher CD36 expression. **a**, Lipid staining of MAMs isolated from normal liver or metastatic liver tumors along with the quantification (*n* = 4). Scale bar, 5 μm. **b** Lipid staining of BMDMs cocultured with or without LLC cells was shown. Lipid dots area were quantified on the right (*n* = 5). Scale bar, 10 μm. **c** Lipid levels in BMDMs were determined by flow cytometry (*n* = 4). **d**, **e** Relative levels of triglyceride (TG) and diglyceride (DG) in BMDMs were determined by LC-MS (*n* = 5). **f** Oxygen consumption rates (OCRs) of the BMDMs (*n* = 3). **g**, **h** Fatty acid uptake in MAMs (**g**, *n* = 4) or BMDMs (**h**, *n* = 5). **i** The MFI of CD36 in MAMs was analyzed by flow cytometry (*n* = 4). **j**, **k** The mRNA expression of CD36 in BMDMs treated with LLC cells or their conditional medium (CM) (*n* = 4). **l** Representative histogram (left) and quantitative results of the MFI of CD36 staining in BMDMs (*n* = 3). **m** Comparison of CD36 expression in CD206^low^ and CD206^high^ subsets of BMDMs (*n*  =  4). Data are representative of two independent experiments with similar results **a**, **b**, **g**, **h**, **i**, **l**, **m**. Values for n represent biologically independent samples. Data are mean ± SEM and *P* values were determined by unpaired two-tailed Student’s t-test. Source data are provided as a Source Data file.

In response to TME, macrophages undergo metabolic reprogramming that subsequently influences their functional phenotypes. MAMs isolated from metastatic liver tumors contained notably more lipid droplets than native macrophages isolated from normal livers (Fig. 3a). In vitro, tumor cell-stimulated BMDMs displayed a typical lipid-load phenotype (Fig. 3b, c and Supplementary Fig. 5a). Lipid analysis revealed significantly increased levels of triglycerides (TG) and diglycerides (DG) in BMDMs that were cocultured with tumor cells (Fig. 3d, e). As a result, tumor-stimulated BMDMs showed enhanced mitochondrial respiration than the controls (Fig. 3f). Importantly, tumor-associated MAMs and BMDMs had a higher ability to uptake LCFAs than their normal counterparts (Fig. 3g, h). Among the membrane fatty acid transporters, CD36 was the most abundant one expressed on macrophages (Supplementary Fig. 5b). As expected, the expression of CD36 was increased in tumor-associated MAMs and BMDMs as compared with native macrophages (Fig. 3i–l and Supplementary Fig. 5c). The expression of other fatty acid transporters, such as *Fatp1-6* was unchanged or decreased by tumor cells (Supplementary Fig. 5d).

It is well-known that macrophages consist of distinct subpopulations with specialized functions in tumors. In IL-4-induced M2 macrophages, both CD36 expression and LCFA uptake were higher than M0 macrophages or LPS-induced M1 macrophages (Supplementary Fig. 5e–h). Further analysis of CD36 expression revealed a higher level in the CD206^high^ subsets versus the CD206^low^ subset of MAMs or BMDMs (Fig. 3m and Supplementary Fig. 5i, j), suggesting CD36 exhibits a unique expression pattern in protumoral M2-like macrophages. Collectively, the above results indicate that CD36-mediated lipid uptake might be a key factor contributing to lipid deposition and subsequently immune dysfunction in MAMs.

### Extracellular vesicle-carrying lipids derived from tumor cells fuel macrophages

**Fig. 4:**
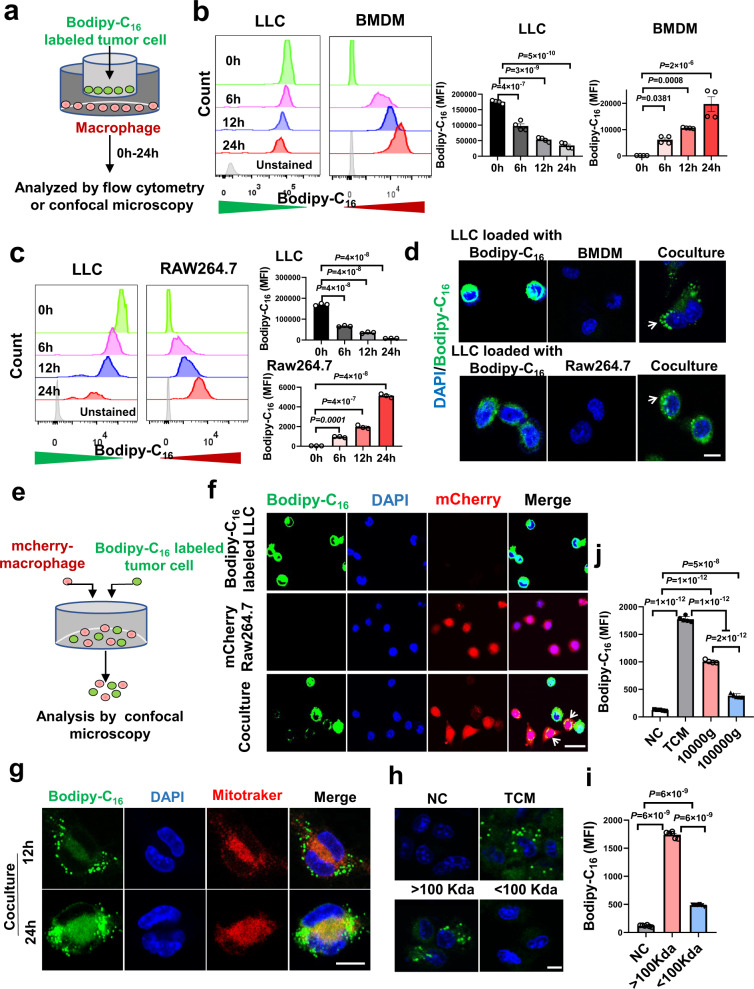
Macrophages take up exogenous fatty acids that released by tumor cells. **a**, Schematic representation of the fluorescent lipid pulse-chase assay. BMDMs **b** or RAW264.7 **c** cells were indirectly cocultured with LLC cells that were preloaded with Bodipy-C_16_ (1 μM). The MFI of Bodipy in these cells was determined by flow cytometry at indicated times (**b**, *n* = 4; c, *n* = 3). **d** Representative images showing the lipid transfer from Bodipy-C_16_-labled LLC tumor cells to macrophages (*n* = 5). Scale bar, 10 μm. **e, f** Bodipy-C_16_-preloaded LLC cells were directly cocultured with mCherry-RAW264.7 cells (*n* = 5). Representative images showing the lipid transfer from LLC tumor cells to RAW264.7 cells. Scale bar, 20 μm. **g** Localization of the incorporated lipids with MitoTracker red in BMDMs (*n* = 5). Scale bar, 10 μm. **h**, **i** Total conditioned medium (TCM) was prepared from Bodipy-C_16_-preloaded LLC cells and separated with a 100 Kda filter. Fluorescent lipids incorporated into BMDMs were detected after incubation with different fractions for 24 h by confocal microscopy (**h**, scale bar, 10 μm) and flow cytometry **i** respectively (*n* = 6). **j** Pellets (10,000 g and 100,000 g) of extracellular vesicles were isolated from Bodipy-C_16_-preloaded LLC cells by ultracentrifugation. BMDMs were incubated with the indicated pellets and then Bodipy intensity was measured after 24 h (*n* = 5, 5, 4, 5 from left to right group). Data are representative of two independent experiments with similar results. Values for *n* represent biologically independent samples. Data are mean ± SEM and *P* values were determined by one-way ANOVA with Dunnett’s **b**, **c** or Tukey’s multiple comparison tests **i**, **j**. Source data are provided as a Source Data file.

A recent study showed that fatty acids are preferentially partitioned into tumor cells, indicating limited accessibility of circulating fatty acids to macrophages^16^. We tested whether tumor cells transfer their stored lipids to macrophages using a lipid pulse-chase assay. Tumor cells were preincubated with a fluorescently labeled LCFA (BODIPY-C_16_) before coculture with macrophages (Fig. 4a). Flow cytometry analysis showed that the fluorescent signal was steadily increased in macrophages, but decreased in tumor cells in a time-dependent manner following coculture, regardless of tumor or macrophage type (Fig. 4b, c and Supplementary Fig. 6a, b). The labeled lipids originally only presented in tumor cells were detected in macrophages and formed lipid droplet-like structures upon direct or indirect coculture (Fig. 4d–f). Following coculture, fluorescent LCFAs primarily localized to lipid droplets appeared to be redistributed into mitochondria in macrophages over time (Fig. 4g). To obtain in vivo evidence of lipid transport from tumor cells to liver MAMs, tumor cells preloaded with BODIPY-C_16_ were intrasplenically injected into mice. By analyzing the isolated cells from livers, we found the fluorescent signal was increased in MAMs after tumor cells arrived (Supplementary Fig. 6c). Together, these results indicate that tumor-derived lipids can be transported into macrophages for oxidation.

We next investigated how tumor cells transfer their stored lipids to macrophages. Lipid trafficking mediated by extracellular vesicles appears as a new mechanism involved in cell-cell communication^17–20^. We divided the tumor conditioned medium (TCM) into two fractions using a 100 Kda filter to roughly distinguish between free fatty acids (<100 Kda) and fatty acid-carrying vehicles (>100 Kda). Surprisingly, the fraction (>100 Kda) induced fluorescent signal in macrophages as effectively as TCM did (Fig. 4h, i). A little fluorescent signal could be detected in macrophages treated with the fraction (<100 Kda). Also, upon incubating with the fraction (>100 Kda), the labeled LCFAs were transported into the mitochondria of macrophages (Supplementary Fig. 6d). The accessibility of tumor-derived lipids to macrophages was largely abolished at 4˚C (Supplementary Fig. 6e, f), suggesting these lipids were mainly carried by vesicles. Additionally, extracellular vesicles were isolated from the TCM via centrifugation. After gradient separation of crude vesicles, we observed that fluorescent signals were detected in macrophages after incubating with the pellets after 10000 g or 100000 g centrifugation (Fig. 4j. Thus, tumor cells transfer their lipids to macrophages mainly through the release of lipid-enriched vesicles.

### CD36 is involved in tumor-induced lipid changes in macrophages

Then we asked whether tumor and normal cells differ in their ability to deliver lipids to the macrophages. Compared with normal cells, including hepatocytes, HEK293T and NIH-3T3, tumor cells exhibited a unique ability to transfer their lipids to macrophages (Supplementary Fig. 6g). Next, we explored the predominant cell population accessing tumor cell-derived lipids in the liver metastasis model. Tumor-infiltrating CD45^+^ immune cells took up fluorescent lipids released from tumor cells, which is higher in myeloid subsets (CD45^+^CD11b^+^) than lymphocyte subsets (CD45^+^CD11b^−^), and highest in macrophages (CD45^+^CD11b^+^F4/80^+^) (Supplementary Fig. 6h). Between isolated macrophages and CD8^+^ T cells incubating TCM, macrophages had significantly higher uptake of tumor-derived fluorescent lipids (Supplementary Fig. 6i). These data suggest that tumor cell-derived lipids are partitioned into macrophages by unknown mediators.

**Fig. 5:**
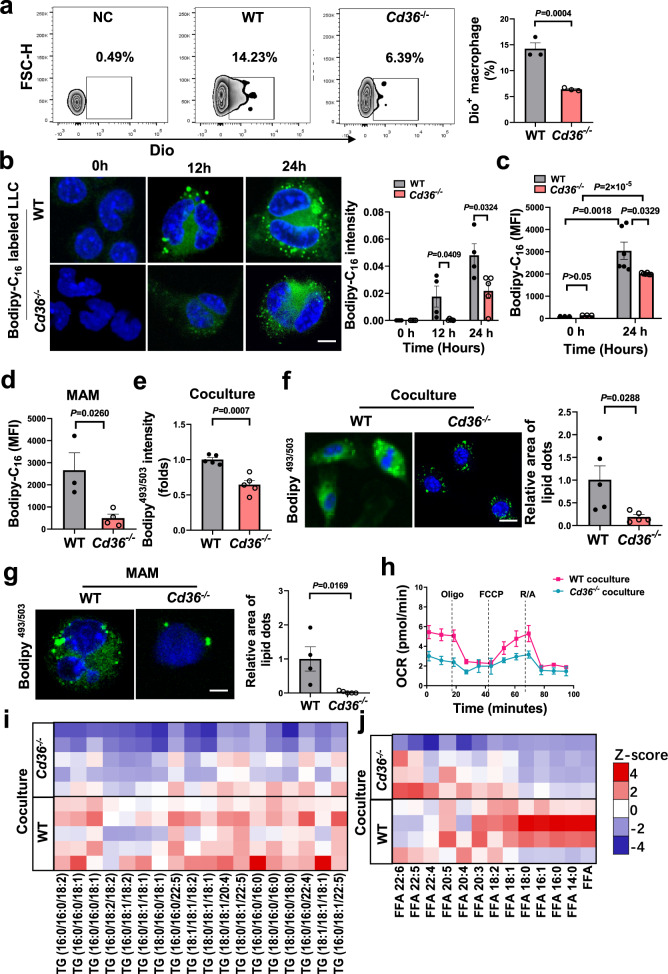
CD36 increases tumor cell-derived fatty acid uptake and lipid deposition in macrophages. **a**, Representative plots of Dio signals in macrophages after intravenous injected with Dio-labled extracellular vesicles for 24 h (*n* = 3). **b**, Representative images showing the engulfment of LLC-derived fluorescent lipids by WT or *Cd36*^−/−^ BMDMs along with quantification. *n* = 4 for WT group, *n* = 5 for *Cd36*^−/−^ group, Scale bar, 5 μm. **c**, The MFI of Bodipy in WT and *Cd36*^−/−^ BMDMs was determined after incubation with Bodipy-C_16_-labled LLC tumor cells (*n* = 3 for 0 h group, *n* = 6 for 24 h group). **d**, The MFI of Bodipy in macrophages after intrasplenically injected with Bodipy-C_16_-labled LLC cells (1 × 10^6^) for 24 h (*n* = 3 for WT group, *n* = 4 for *Cd36*^−/−^ group). **e**, Lipid levels in WT and *Cd36*^−/−^ BMDMs cocultured with LLC cells (*n* = 5). **f**, Lipid staining of WT and *Cd36*^−/−^BMDMs cocultured with LLC cells (*n* = 5). Scale bar, 10 μm. **g**, Lipid staining of WT and *Cd36*^−/−^ MAMs (*n* = 4 for WT group, *n* = 5 for *Cd36*^−/−^ group). Scale bar, 5 μm. **h**, OCRs of the WT and *Cd36*^−/−^ BMDMs cocultured with LLC cells (*n* = 3). **i**, Lipidomic analysis of TG molecular species in WT and *Cd36*^−/−^ BMDMs (*n* = 5). **j**. Free fatty acid analysis of WT and *Cd36*^−/−^ BMDMs (*n* = 4). Data are representative of two independent experiments with similar results (**a**–**c**, **f**, **g**). Values for n represent biologically independent samples. Data are mean ± SEM and P values were determined by one-way ANOVA with Tukey’s multiple comparison tests (**a**, **b**) or unpaired two-tailed Student’s t-test (**c**–**f**). Source data are provided as a Source Data file.

The pattern of CD36 expression in tumor-infiltrating immune cell subsets correlated with the pattern of tumor-derived lipids uptake by those cells (Supplementary Figs. 6h and  2g), proposing a possibility of CD36 in mediating lipid-enriched vesicles uptake. CD36 can interact with phosphatidylserine and mediate the engulfment of vesicles^21–23^. Here, we found that, in vivo, tumor-derived extracellular vesicles were transferred less efficiently to macrophages that are CD36-deficient (Fig. 5a). Among the separated immune cells, their ability to take up tumor-derived lipids was positively related to CD36 expression (Supplementary Fig. 6j). Notably, the internalization of tumor-derived lipids by macrophages was markedly decreased when CD36 was absent in vitro (Fig. 5b, c). Additionally, the transfer of BODIPY-C_16_ from cancer cells to MAMs was markedly decreased when CD36 was absent in vivo (Fig. 5d). We speculated that reduced access to tumor-derived lipid would mitigate lipid deposition. As expected, the blockade of CD36 inhibited lipid deposition in tumor-treated macrophages (Fig. 5e–g). In accordance, mitochondrial respiration was decreased in tumor-treated macrophages from *Cd36*^−/−^ mice (Fig. 5h).

To obtain a more comprehensive understanding of the role of CD36 in regulating lipid metabolism, we carried out an analysis of lipid metabolites in WT and *Cd36*^−/−^ BMDMs using untargeted LC-MS. Tumor-treated BMDMs from *Cd36*^−/−^ mice showed a distinct lipidomic profile compared with those from WT mice (Supplementary Fig. 7a–c). The down-regulated lipids were mainly enriched in the major lipid class of glycerolipids, in which TG was notably decreased (Fig. 5i and Supplementary Fig. 7d–f). The content of other lipid classes, including ceramides phosphate (CerP), phosphatidylcholine (PC), and Sphingosine (So) was also significantly decreased (Supplementary Fig. 7d). By analyzing LCFA profile based on GC-MS, a substantially reduced abundance of LCFAs was observed in tumor-treated macrophages from *Cd36*^−/−^ mice, with a greater fold reduction in saturated and mono-unsaturated fatty acid species (Fig. 5j and Supplementary Fig. 7g, h), indicating that the lipidomic alterations observed in *Cd36*^−/−^ macrophages might be attributed to defect uptake of tumor-derived LCFAs. Altogether, these above results indicate that CD36 is a critical regulator for the internalization of tumor-derived lipids, that may be responsible for the orchestration of lipid metabolism in tumor-related macrophages.

### Tumor-derived lipids drive macrophage M2 polarization through CD36

**Fig. 6:**
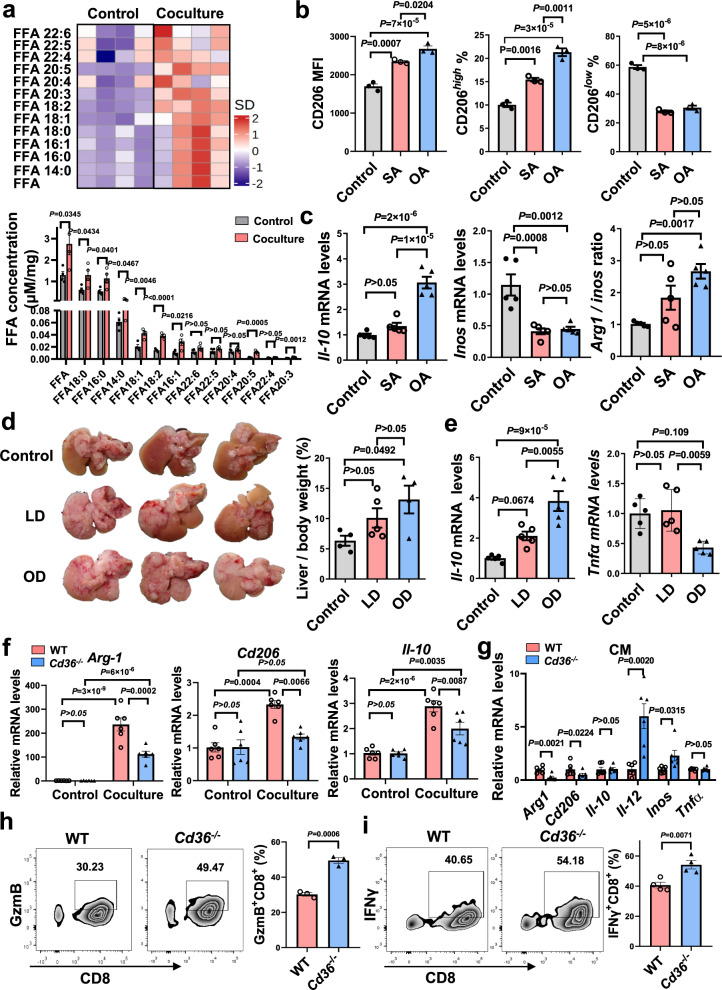
Blockade of CD36 reduces macrophage M2 polarization. **a**, Free fatty acid analysis of BMDMs treated with or without LLC cells (n = 4). **b**, The MFI of CD206 surface staining, and percentages of CD206-expressing cells in BMDMs treated with OA (10 μM) or SA (10 μM) (n = 3). **c**, The mRNA levels of indicated cytokines in RAW264.7 cells treated with OA or SA (n = 5). **d**, Liver metastasis formation in mice fed with a high olive diet (OD, 45% kcal from fat) or a high lard diet (LD, 45% kcal from fat) after intrasplenic injection of LLC cells. The percentage of liver to body weight was shown on the right (n = 4, 5, 4 from left to right group). **e**, The mRNA levels of *Il-10* and *Tnfα* in metastatic tumors from the OD- or LD-fed mice (n = 5). **f**, The mRNA levels of indicated cytokines in WT and *Cd36*^−/−^ BMDMs cocultured with or without LLC cells (n = 6). **g**, The mRNA levels of indicated cytokines in WT and *Cd36*^−/−^ BMDMs treated with the CM from LLC cells (n = 6). **h, i**, After BMDMs were treated with tumor cell CM, CD8^+^ T cells were cocultured with BMDMs (10:1) for 72 hours, and the production of GzmB **(h**, n = 3**)** and IFN-γ **(i**, n = 4**)** in CD8^+^ T cells were assessed. Data are representative of two independent experiments with similar results (**f**–**i**). Values for n represent biologically independent samples. Data are mean ± SEM and P values were determined by one-way ANOVA with Tukey’s multiple comparison tests (**b**–**f**) or unpaired two-tailed Student’s t-test (**g**–**i**). Source data are provided as a Source Data file.

We asked whether tumor-derived lipids would promote the conversion of macrophages to a tumor-promoting phenotype. GC-MS analysis of the liver metastasis-bearing mice revealed an abundance of LCFAs in the TME, commonly saturated and mono-unsaturated LCFAs (Supplementary Fig. 8a). In accordance with this, tumor-cocultured macrophages showed elevated levels in saturated and mono-unsaturated LCFAs (Fig. 6a). So, we evaluated the effects of stearic acid (SA, 18:0) and oleic acid (OA, 18:1) on macrophage function. SA or OA treatment enhanced macrophage M2 activation, with a greater change observed in the OA-treated group (Fig. 6b, c). Coculture of OA-treated BMDMs significantly dampened the expression of GzmB and IFNγ of CD8^+^ T cells (Supplementary Fig. 8b, c), suggesting an immunosuppressive phenotype of macrophages was induced by LCFAs. Similar to in vitro observations, dietary fat supplementation increased the expression of M2 markers and accelerated the development of mouse liver metastasis (Fig. 6d, e and Supplementary Fig. 8d). A trend toward aggravated liver metastasis was noted in mice fed an olive oil diet (OD), that enriched in OA, than mice fed a lard diet (LD). Thus, these data indicate that the acquisition of tumor cell-derived fatty acids, especially mono-unsaturated LCFAs, induces macrophage activation toward an M2-like phenotype.

Then we examined the effects of CD36 on the polarization of macrophages. Flow cytometry analysis of tumor-treated BMDMs showed a decreased percentage of antitumor M1-type macrophages (CD206^low^CD80^high^), but an increased presence of protumor M2-type macrophages (CD206^high^CD80^low^), which could be largely abrogated by CD36 deletion (Supplementary Fig. 8e, f). *Cd36*^−/−^ BMDMs expressed lower M2 markers (*Arg1, Cd206, Il-10*) but higher M1 markers (*Il-12, Tnfα)* compared with WT BMDMs upon incubation with tumor cells or TCM (Fig. 6f, g and Supplementary Fig. 8g). The suppressive effects of tumor-treated *Cd36*^−/−^ BMDMs on CD8^+^ T cells were reduced when compared with WT BMDMs, as demonstrated by increased GzmB and IFNγ expression of CD8^+^ T cells (Fig. 6h, i). In addition, *Cd36*^−/−^ BMDMs suppressed response to IL-4-induced M2 activation, but promoted IFNγ-induced M1 activation (Supplementary Fig. 8h, i). Then we evaluated the role of CD36 in mediating MAM polarization in vivo. Analysis of gene expression in metastatic liver tissues showed that M1 markers were increased, but M2 markers were decreased in *Cd36*^−/−^ mice compared with WT mice (Supplementary Fig. 8j). Taken together, these results suggest that macrophages are endowed with a tumor-promoting phenotype after engulfing lipids from tumor cells through a CD36-dependent mechanism.

### CD36 reshapes the liver immune microenvironment

**Fig. 7:**
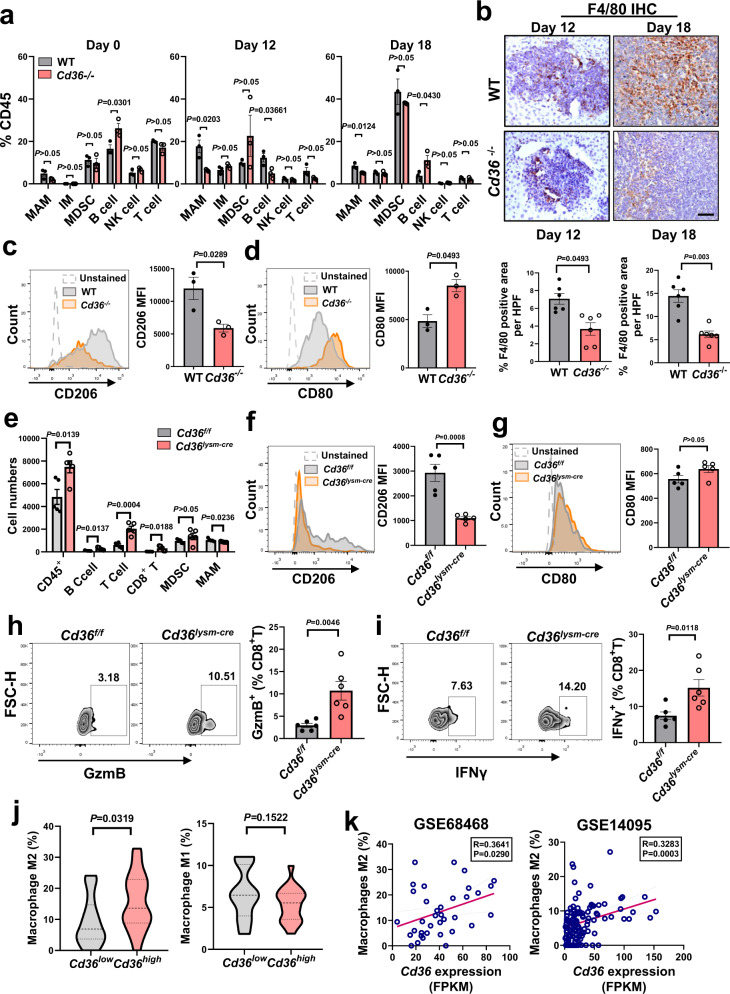
Effects of CD36 on tumor immunity. **a**, Percentages of indicated cell populations among CD45^+^ cells in metastatic liver tumors or normal livers of WT and *Cd36*^−/−^ mice (*n* = 3). **b**, Immunohistochemistry staining of F4/80 in the liver sections from the WT and *Cd36*^−/−^ mice at indicated times along with the quantification (*n* = 6). Scale bar, 100 μm. **c**, **d**, Representative histogram (left) and quantitative results of the MFI of CD206 or CD80 staining in MAMs on day 18 (*n*  =  3). **e**, Numbers of indicated cell populations in the metastatic liver tumors of WT and *Cd36*^*lysm-cre*^ mice on day 12 (*n* = 5). **f**, **g**, Representative histogram (left) and quantitative results of the MFI of CD206 or CD80 staining in MAMs (*n*  =  5). **h**, **i**, CD8^+^ T cells were isolated from the metastatic liver tumors of WT and *Cd36*^*lysm-cre*^ mice and the production of GzmB **(h**, *n* = 6**)** and IFN-γ **(i**, *n* = 6**)** was assessed. **j**, M1 or M2-type macrophage numbers in *Cd36*^low^ (*n* = 18) and *Cd36*^high^ (*n* = 18) expression groups in patients with liver metastasis (GSE68468). **k**, The correlation of CD36 with M2-type macrophage infiltration in patients with liver metastasis from two GEO datasheets (*n* = 36, 116 respectively). *P* value was calculated by Pearson correlation analysis. Values for n represent biologically independent samples. Data are mean ± SEM and *P* values were determined by unpaired two-tailed Student’s t-test (**a**–**g**). Source data are provided as a Source Data file.

We seek to gain further insight into the function of CD36 in liver TME. The immune cell profiling did not differ significantly between normal WT and *Cd36*^−/−^ livers, except for an increase in B cells in *Cd36*^−/−^ livers (Fig. 7a and Supplementary Fig. 9a). After tumor cell implantation, MAMs were the predominant population infiltrated on day 12, while myeloid-derived suppressor cells (MDSCs) were the prominent component on day 18, but the frequency of T cells, B cells and NK cells decreased over time (Fig. 7a). Compared with the WT mice, *Cd36*^−/−^ mice contained a significantly decreased percentage of MAMs both on day 12 and 18, and an increased rate of B cells on day 18 (Fig. 7a). Immunohistochemical staining demonstrated diminished MAMs in metastatic foci of *Cd36*^−/−^ mice (Fig. 7b). Immunofluorescence staining of hepatic metastatic tumors showed that F4/80^+^/CD206^+^ double positive macrophage subpopulation was more abundant in WT mice than in *Cd36*^−/−^ mice (Supplementary Fig. 9b). Flow cytometry analysis also demonstrated down-regulated CD206 expression and increased CD80 expression of MAMs in the *Cd36*^−/−^ mice (Fig. 7c, d and Supplementary Fig. 9c).

To confirm that, tumor-infiltrating immune cells isolated from *Cd36*^*lysm-cre*^ mice and their littermates were evaluated. We observed that *Cd36*^*lysm-cre*^ mice with LLC liver metastasis showed a diminished frequency of MAMs, but increased frequency of B cells and T cells, including cytotoxic CD8^+^T cells (Fig. 7e). Analysis of the immune cell profile in the B16F10 liver metastasis model yielded similar results (Supplementary Fig. 9d). Further analysis of MAMs from *Cd36*^*lysm-cre*^ mice revealed down-regulated CD206 expression compared with those from control mice, indicating a reduced M2-like feature (Fig. 7f, g and Supplementary Fig. 9e, f). Furthermore, CD8^+^ T cells isolated from *Cd36*^*lysm-cre*^ mice showed significantly increased expression of GzmB and IFNγ (Fig. 7h, i). These results suggest that deletion of CD36 in MAMs may enhance CD8^+^T cell immunity to achieve antitumor effects.

Having demonstrated a crucial role of CD36 in mouse liver TME, we reached out to verify the clinical significance of CD36 in human hepatic metastasis. We observed that patients with higher CD36 expression had an increased proportion of M2-type MAMs in the GES68468 dataset (Fig. 7j and Supplementary Fig. 9g, h). In another human liver metastasis dataset GSE14095, an increased proportion of M2-type MAMs and neutrophils, and decreased CD4^+^ T cells and follicular helper T cells were found in patients with higher CD36 levels (Supplementary Fig. 10a). There was a significant positive correlation between CD36 expression and M2-type MAMs infiltration in patients with liver metastases (Fig. 7k). Additional gene expression analysis revealed that *Cd36* expression was positively correlated with M2-type markers, such as *Arg1*, *Cd206* and *Cd163* in patients with metastases (Supplementary Fig. 10b, c). We then investigated the biomarker potential of CD36 in liver cancer patients. There was increased plasma sCD36 in liver cancer patients with metastases compared to those without (Supplementary Fig. 10d). Besides, CD36 expression on PBMCs was positively correlated with the incidence of liver cancer metastasis (Supplementary Fig. 10e, f). These data suggest that CD36 is associated with the immunosuppression pattern of liver metastasis in mice and patients, indicating a potential marker for the progression of liver metastasis.

## Discussion

**Fig. 8:**
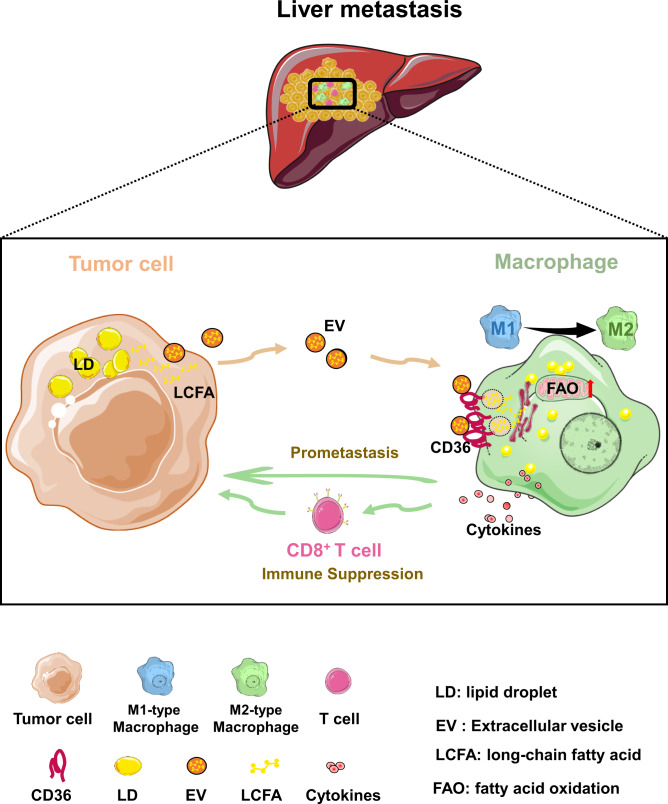
The proposed model of CD36-mediated metabolic crosstalk between tumor cells and macrophages. The graphical abstract describes how tumor cells reprogram the metabolism and immunity of MAMs by releasing lipid-carrying vesicles and increasing CD36, and further how this process involves in liver metastasis.

Exploiting the mechanisms by which TME factors drive immune suppression is an attractive strategy for improving cancer immunotherapy. In this study, we uncover that tumor-related lipid metabolites influence the composition of TME and the function of intratumor macrophages. Our results suggest that tumor-derived lipids functionally reprogram macrophages through the upregulation of CD36 (Fig. 8). Targeting the tumor-macrophage metabolic interface via CD36 inhibition may be an effective treatment strategy against liver metastasis.

Immune cells accumulate lipids in response to TME, a feature that significantly contributes to immune dysfunction. Several studies suggested that major components in the TME, including Treg cells, MDSCs, and macrophages, have a higher ability to uptake lipids^9,24,25^; however, none of these studies has elucidated the source of lipids in the TME and how these lipids are carried. Here, we observed that LCFAs secreted by tumor cells shape a lipid-enriched TME. We detected higher concentrations of saturated and monounsaturated LCFAs in the TME of mice bearing liver metastasis. Consistently, a recently published study by Xu et al. has found higher amounts of fatty acids in tumor interstitial fluid^26^. More interestingly, our results demonstrated that the LCFAs released by tumor cells were delivered by extracellular vesicles. Extracellular vesicles are emerging as a vesicular lipid transporter involved in cell-to-cell communication^18^. For example, lipid-filled vesicles were shown to mediate lipid transport from adipocytes to macrophages or tumor cells^19,20^. In addition, tumor-derived fatty acid-carrying exosomes induce a metabolic shift toward oxidative phosphorylation in dendritic cells^10^. Our data showed that tumor cells exhibited a unique ability to release LCFA-carrying extracellular vesicles, which are preferentially ingested by macrophages. Acquisition of lipids from tumor cells finally impaired the antitumor functions of macrophages. Our findings highlight the importance of tumor-derived lipid metabolites in defining the immunosuppressive TME, and improve our understanding of the role of lipid-rich TME in regulating tumor immunity.

The scavenge receptor CD36, also identified as a fatty acid translocase, is a central regulator of both immune and metabolic pathways^27–29^. An emerging role of CD36 in cancer has been proposed in recent years. CD36-driven fatty acid metabolism contributes to the growth and metastasis of multiple cancers in a cancer cell-intrinsic manner^14,30–34^. In addition, several studies suggested CD36-mediated lipid metabolism functions in the TME, leading to immune tolerance and tumor growth. A recent report by Wang et al. showed that intratumoral Treg cells rely on CD36 to facilitate fatty acid uptake, which supports mitochondrial biogenesis and the suppressive function of Treg cells^24^. Likewise, Ma et al. and Xu et al. found that CD36 involved in arachidonic acid or ox-LDL induced ferroptosis and dysfunction of CD8^+^T cell^26,35^. In this study, we found that CD36 was predominantly expressed in the MAMs among subsets of metastatic liver tumors. Our findings identify a role of CD36 in mediating the internalization of tumor-derived lipid-carrying vesicles by MAMs, which is beyond its general function as a fatty acid transporter. Tumor-derived lipids uptake via CD36 subsequently triggered the orchestration of lipid metabolism in MAMs. This lipid remodeling in MAMs shaped their tumor-promoting phenotype with immunosuppressive activities, mainly manifested by suppression of CD8^+^ T cell function. The absence of CD36 in MAMs reduced their M2 polarization and also restored CD8^+^ T cell immunity. Our data in this study delivers insights into the specific field of macrophage fatty acid metabolism and the broader field of tumor immunology.

Macrophages in the liver can be broadly categorized into two classes: embryonically derived tissue-resident macrophages known as Kupffer cells, and infiltrating macrophages derived from monocytes that originate from bone marrow^36^. A highly debated question is whether macrophage origin dictates functionality in the context of cancer. Using single-cell RNA-sequencing of hepatic mononuclear cells isolated from metastatic liver tumors, Yu et al. identified that monocyte-derived macrophages were the main macrophage population and exhibited an immunosuppressive gene signature^1^. Consistently, Nielsen et al. found that, in liver metastasis, MAMs originate from inflammatory monocytes and support the metastatic growth of disseminated cancer cells^36^. Thus, we speculate that monocyte-derived macrophages may be crucial for CD36-mediated liver metastasis, but it still needs to be further investigated.

Patients with liver metastases are strongly associated with poor prognosis and diminished therapeutic responsiveness. The management of liver metastasis is far from satisfactory and remains a therapeutic challenge. Here, we elucidate how tumor cells drive metabolic and functional remodeling of macrophages to promote their own progress, which is effective in liver metastasis and may extend to other types of tumors. We highlight the importance of lipid-enriched vesicles released by tumor cells and the upregulated expression of CD36 in macrophages in such tumor-macrophage metabolic crosstalk. In addition, blockade of CD36 in MAMs restores CD8^+^T cell immunity and ameliorates liver metastasis in preclinical mouse models. Our work suggests that targeting CD36 on MAMs helps to relieve tumor-macrophage metabolic interdependence and associated immune suppression, which may be an appealing therapeutic strategy against liver metastasis.

## Methods

### Human samples

A total of 75 blood samples of liver cancer patients were collected from the Second Affiliated Hospital of Chongqing Medical University. The plasma concentrations of soluble CD36 (sCD36) were determined using an ELISA kit (#E0674h, EIAab Science). Peripheral blood mononuclear cells were isolated using a commercial kit (#P5230, Solarbio). Participating patients all provided written informed consent. The study was approved by the Ethics Committee of the Second Affiliated Hospital of Chongqing Medical University.

### Mice

CD36 knockout (*Cd36*^−^^/−^) mice created on a C57BL/6 J background were kindly provided by Dr. Maria Febbraio (Lerner Research Institute). Alb-cre^+/−^ mice and Lysm-cre^+/+^ mice were obtained from Shanghai Research Center for Model Organisms (China). Cd36^f/f^ mice, in which the exon 5 of the CD36 allele was flanked with loxP recombination sites were generated and crossed with either Alb-cre^+/−^ or Lysm-cre^+/+^ mice to generate hepatocyte- or myeloid-specific CD36 knockout mice^37^. Cd36^f/f^ mice were identified by tail genomic DNA analysis with primer F specific to the upstream LoxP locus (5′-TCCCTTGAATTGGCCAACTTTG-3′) and primer R (5′-ACTGCCTGTGAGAACTTCTCAA−3′), an antisense specific to the downstream LoxP locus. CD36 deletion was confirmed by mRNA and protein analysis. Mice were maintained in a controlled environment of 20–23 °C, with a 12/12 h light/dark cycle, 50–60% humidity, and food and water provided ad libitum. Male, 8–10 weeks old mice as described above were used for this study. Animal care and experimental procedures were performed with approval from the animal care committees of Chongqing Medical University.

### Cell lines

Murine Lewis lung carcinoma (LLC) cell line, melanoma cell line B16F10 and hepatoma cell line Hepa1-6 were purchased from the Cell Bank of Chinese Academy of Sciences (Shanghai, China). The macrophage cell line RAW264.7 was kindly provided by Dr. JH. Yan (Chongqing Medical University, China). Colon carcinoma cell line CT26 was purchased from ATCC (Rockville, MD, USA). All cells were cultured in RPMI-1640 growth medium containing 10% fetal bovine serum, 100 U/mL penicillin, and 100 mg/mL streptomycin.

### Liver metastasis model

A total of 1 × 10^6^ tumor cells in 100 μl PBS or PBS alone were inoculated into the spleen of the anesthetized mice. Then an immediate splenectomy was performed^1^. Mice were euthanized at day 12 or day 18 post-inoculation or when they experienced lost >15% of their total body weight or lack of mobility. The livers were removed and metastatic tumor burden was assessed by quantifying the number and size of metastatic foci. For evaluation of the retention of tumor cells in the liver, CellTracker Green CMFDA-labeled tumor cells were inoculated, and liver sections were imaged by fluorescence microscopy at 1 h, 12 h, and 24 h postinoculation.

### Preparation of bone marrow-derived macrophages (BMDMs)

Bone marrow cells were isolated from the tibia and femur of 6-8-week-old mice and differentiated in the presence of M-CSF (10 ng/mL) in RPMI-1640 growth medium. On day 5, coculture systems were established by using transwell inserts, in which LLC cells were loaded in the upper insets, and BMDMs were seeded in the lower compartment. Following 2 days coculture, tumor-educated macrophages were harvested and analyzed. In some experiments, differentiated BMDMs were polarized into M1 or M2 phenotypes in the presence of IFN-γ (10 ng/mL) or IL-4 (10 ng/mL) for 24 h.

### Cell isolation

The freshly isolated liver tumor tissues or normal liver tissues were cut into pieces and digested in Hanks’ buffer containing IV collagenase (Sigma) for 30 min at 37°C. The dissociated cells were filtrated through a 70 μm nylon mesh and centrifuged at 50 g for 5 min to remove hepatocytes. The suspension was centrifuged at 800 g for 5 min, and then the pellets were resuspended in red blood cell lysis buffer. Cells were recovered by centrifugation at 800 g, and used for further analysis of liver immune cells.

Single-cell suspensions from murine spleens were prepared by mechanical disruption in Hanks’ buffer. Cells in suspension were centrifuged for 5 min at 400 g. After red blood cell lysis, cells were filtrated through a 70 µm nylon mesh. CD8^+^ T cells were isolated from spleen cells using magnetic bead sorting according to manufacturer’s instructions (#480008, Biolegend). Spleen macrophages were allowed to adhere for 2 h at 37 °C and the suspension cells were washed extensively with warm medium prior to use.

### Flow cytometry and cell sorting

Cells were incubated with Fc block antibody (#14-0161-82, eBioscience, 1:100) for 30 min at 4 °C to prevent nonspecific binding. Cell surface labeling was conducted with fluorescently conjugated antibodies for 30 min at 4°C. The following antibodies were used: BV510 anti-mouse CD45 antibody (#103137, Biolegend, 1:25), PerCP anti-mouse CD11b (#101229, Biolegend, 1:50), APC anti-mouse F4/80 (#17-4801-80, eBioscience, 1:25), PE/Cy7 anti-mouse Gr1 (#108415, Biolegend, 1:100), FITC anti-mouse CD3 (#11-0032-82, eBioscience, 1:50), PE/Cy7 anti-mouse CD19 (#25-0193-81, eBioscience, 1:50), BV421 anti-mouse NK1.1 (#108741, Biolegend, 1:20), PE anti-mouse CD36 (#562702, BD Biosciences, 1:100), PE anti-mouse CD4 (#4329629, Invitrogen, 1:200), APC anti-mouse CD8a (#17-0081-81, eBioscience, 1:200), BV421 anti-mouse CD206 (#141717, Biolegend, 1:25), FITC anti-mouse CD80 (#FITC-65076, Proteintech, 1:200), FITC anti-mouse GzmB (#372206, Biolegend, 1:25), FE anti-mouse IFNγ (#PE-65153, Proteintech, 1:50). Fluorescence data were collected using a FACSAria II flow cytometer (BD Biosciences, USA) and analyzed employing a FlowJo software. The single cell population was separated with a BD FACSAria II Cell Sorter.

### T cell stimulation assay

CD8^+^ T cells isolated from spleen cells were cocultured with BMDMs at 10:1 in the presence of anti-CD3 (5 μg/ml, #553057, BD Pharmingen) and anti-CD28 (2 μg/ml, #553294, BD Pharmingen). After 72 h, cells were collected for the determination of GzmB and IFN-γ by flow cytometer.

### Cellular energy metabolism analysis

Cellular energy metabolism was measured by the Seahorse XFe24 Analyzer (Agilent, USA) as described previously^27^. In brief, the tumor cell-cocultured macrophages or the control macrophages (2 × 10^4^) were planted in XFe24 microplates the night before the experiment. Oxygen consumption rates (OCR) were determined after the sequential addition of oligomycin (1 μM), FCCP (1 μM) and antimycin/rotenone (1 μM).

### Untargeted lipidomics

BMDMs cocultured with or without tumor cells were resuspended in 1 mL of a mixture of CHCl3: MeOH (2:1). The lipid extract was dried under nitrogen and resuspended in 200 µL water and 240 µL methanol. Then 800 µL of methyl-butyl ether (MTBE) was added and the mixture was ultrasound 20 min at 4 °C. The solution was centrifuged at 14, 000 g for 15 min at 10 °C and the upper organic fraction was obtained, dried under nitrogen and followed by reconstitution in 200 µL 90% isopropanol/acetonitrile. For LC-MS untargeted lipidomics, the extracts were injected into UHPLC Nexera LC-30A coupled to a Q-Exactive mass spectrometer (Thermo Scientific^TM^). Lipid species were identified using the LipidSearch software version 4.2 (Thermo Scientific™). After normalization and integration using the Perato scaling method, the processed data was imported into SIMPCA-P 16.1 (Umetrics, Umea, Sweden) for multivariate statistical analysis, including partial least squares discriminant analysis (PLS-DA). Lipids with significant differences were identified based on a combination of VIP > 1 and *P*-value < 0.05.

### Free fatty acid analysis

Fatty acids were extracted from cells or culture medium using a modified version of the Bligh and Dyer’s protocol. Free fatty acids (C14-C22) were analyzed using an Agilent 1290 UPLC coupled with a triple quadrupole/ion trap mass spectrometer (6500 Plus Qtrap; SCIEX). Lipids were separated by normal phase-HPLC was carried out using a Phenomenex Luna 3 µm-silica column (internal diameter 150 × 2.0 mm) with the following conditions: mobile phase A (chloroform: methanol: ammonium hydroxide, 89.5:10:0.5) and mobile phase B (chloroform: methanol: ammonium hydroxide: water, 55:39:0.5:5.5). Free fatty acids were quantitated using d31-16:0 (Sigma-Aldrich) and d8-20:4 (Cayman Chemicals) as internal standards.

### Lipid transfer assay

Tumor cells were serum starved for 12 h, and then were pre-incubated with BODIPY-C_16_ (#D3821, Invitrogen, 1 µM) at 37 °C for 4 h. The cells were washed three times with PBS containing 0.2% fatty acid-free BSA. After washing, labeled tumor cells were added to the upper chamber, where macrophages had been seeded in the lower chamber of the transwell. In another experiment, labeled tumor cells were cocultured with mCherry-RAW264.7 in a direct cell–cell contact manner. The cells were analyzed at indicated times by flow cytometry or imaged with confocal microscopy.

To detect in vivo lipid transport from tumor cells to macrophages, BODIPY-C_16_-labeled tumor cell were intrasplenically injected into C57BL/6 J mice. After 24 h, the livers were dissected and the fluorescent signal in macrophages was analyzed by flow cytometry.

### Lipid droplet staining

Lipid droplets were stained with Bodipy^493/503^ (Invitrogen). Briefly, fixed cells were incubated with a concentration of 0.2 μg/mL Bodipy^493/503^ solution in the dark for 30 min at 37 °C. The cells were washed and counterstained with DAPI to reveal nuclei. Then, the stained cells were visualized using a confocal microscopy (Leica, Germany).

### Fatty acid uptake assay

To assess the uptake of fatty acid, macrophages were incubated with fluorescent probe BODIPY-C_16_ for 1 h. The uptake of fluorescent fatty acid in cells was examined by flow cytometry.

### Extracellular vesicle isolation

Extracellular vesicles were isolated from fresh cell culture medium by differential centrifugation. In brief, after two steps at 500 g and 3000 g at 4 °C to remove cells and debris, the supernatant was centrifuged at 10,000 g for 30 min at 4 °C to produce the microvesicles-enriched pellets. The resulting supernatant was then filtered through a 0.22 μm filter and centrifuged at 100,000 g for 70 min to produce exosome-enriched pellets. For in vivo trafficking, Dio-labeled pellets were delivered to recipient mice through injection into the tail vein. After 24 h injection, the liver cells were isolated for detecting the appearance of fluorescence.

### Histology

H&E staining, immunohistochemistry and immunofluorescence analysis have been described in our previous study^28^. The following primary antibodies were used: anti-CD31 (#77699, CST, 1:200), anti-Ki67 (#27309-1-AP, Proteintech, 1:2000), anti-F4/80 (#ab6640, Abcam, 1:200) and anti-CD206 (#abs125294, Absin, 1:1000). Quantitative image analysis was performed using Image J 1.47.

### Reverse transcriptase PCR (RT-PCR)

Total RNA was extracted using TRIzol reagent (Takara) and reverse transcribed into cDNA. Next, the cDNA products were subjected to 2-step PCR amplification using the CFX Connect TM real-time system (Bio-Rad, USA). The relative expression of the genes was analyzed using the 2-ΔΔCt method, and β-actin was used as the internal reference gene. Primers used in this study are summarized in Supplementary Table 1.

### Statistical analysis

Values are reported as the mean ± SEM. Statistical analyses were performed using GraphPad Prism 5. Unpaired two-tailed Student’s t-test was used when only two groups were compared, and one-way ANOVA followed by Turkey’s multiple comparison test was used for three or more groups. *P* < 0.05 was considered statistically significant.

### Reporting summary

Further information on research design is available in the Nature Research Reporting Summary linked to this article.

## Supplementary information

Supplementary Information

Reporting Summary

## Data Availability

The single-cell sequencing data are obtained from the Human Protein Atlas^38^ (https://www.proteinatlas.org/). *Cd68* or *Cd36* gene expression in different single cell type clusters of the liver was available from v21.1 proteinatlas.org (https://www.proteinatlas.org/ENSG00000129226-CD68/single+cell+type/liver or https://www.proteinatlas.org/ENSG00000135218-CD36/single+cell+type/liver). *Cd14* or *Cd36* expression in different single cell type clusters of peripheral blood mononuclear cells (PBMCs) was available from v21.1 proteinatlas.org (https://www.proteinatlas.org/ENSG00000170458-CD14/single+cell+type/PBMC or https://www.proteinatlas.org/ENSG00000135218-CD36/single+cell+type/PBMC). RNA-seq data are accessible at the Gene Expression Omnibus (GEO) under accession numbers: GSE68468, GSE14095, GSE41258. All other relevant data supporting the key findings of this study are available within the article, Supplementary Information, or Source Data file. Source data are provided with this paper.

## Source data

Source Data
