# Supplementary material for: CD36-mediated metabolic crosstalk between tumor cells and macrophages affects liver metastasis
Source: Nat Commun. 2022 Oct 2;13:5782. doi: 10.1038/s41467-022-33349-y (PMC9527239; doi:10.1038/s41467-022-33349-y)
Supplement: Supplementary file 1 — Supplementary Information [file 41467_2022_33349_MOESM1_ESM.pdf]

Supplementary Information

CD36-mediated metabolic crosstalk between tumor cells and macrophages affects liver metastasis

Yang et al.

Supplementary Fig. 1

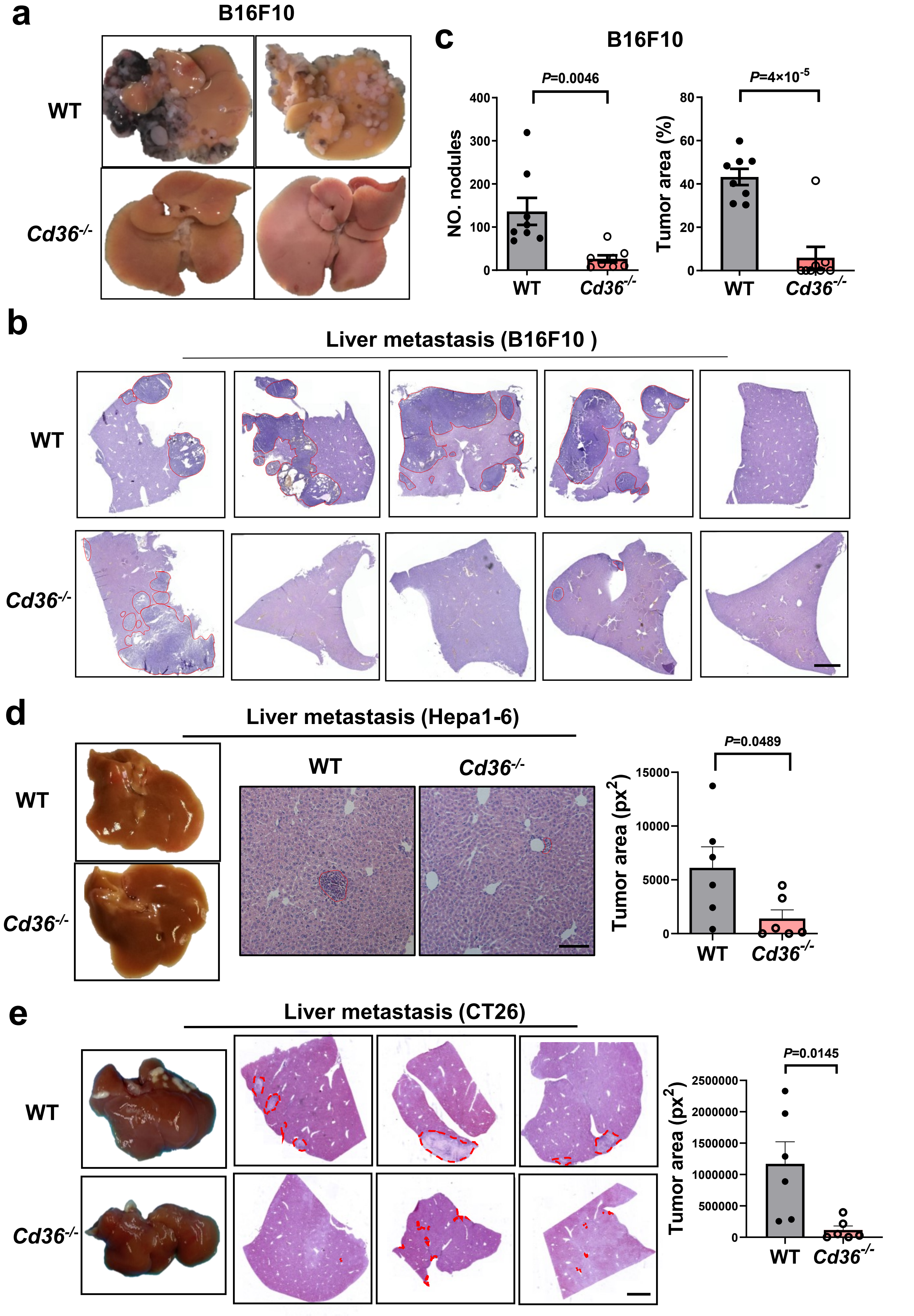

Supplementary Fig. 1 Host CD36 expression contributes to the progression of liver metastasis. **a,** 1×10^6 B16F10 cells were intrasplenically injected into WT and *Cd36*$^{-/-}$ mice (n=8). Macroscopic images of the liver were shown on day 14 postinjection. **b,** HE-stained liver sections from WT and *Cd36*$^{-/-}$ mice. Scale bar, 1000 μm. **c,** The number of metastatic tumors (left), and tumor area (right) were quantified (n=8). **d, e,** 1×10^6 Hepa1-6 (d) or CT26 (e) cells were intrasplenically injected into WT and *Cd36*$^{-/-}$ mice (n=6). Representative liver images (left), HE staining liver sections (middle) along with quantification of tumor area (right) of WT and *Cd36*$^{-/-}$ mice were shown. Scale bar, 200 μm (d) or 1000 μm (e). Data are representative of two independent experiments with similar results (a-d). Values for n represent biologically independent samples. Data are mean ± SEM and P values were determined by unpaired two-tailed Student's t-test. Source data are provided as a Source Data file.

Supplementary Fig. 2

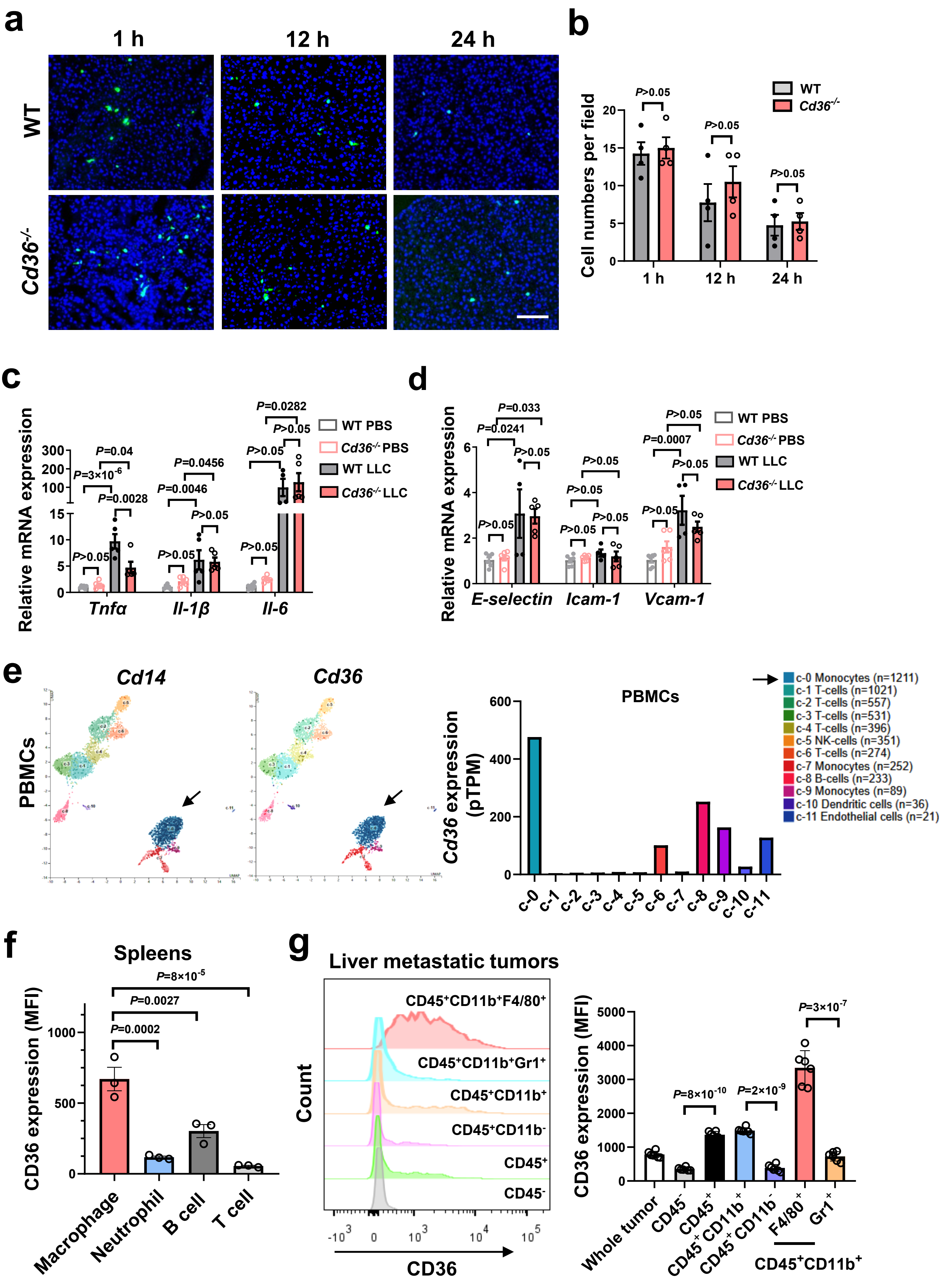

Supplementary Fig. 2 CD36 is predominantly expressed in macrophages.

a, WT and *Cd36*$^{-/-}$ mice were intrasplenically injected with CellTracker Green CMFDA-labeled LLC cells (1×10^6). The liver was excised at the indicated times postinjection and imaged by microscopy. Scale bar, 200 μm. **b,** The number of tumor cell arrest in the livers was quantified (n=4). **c, d,** LLC cells or PBS were injected into the spleen of WT and *Cd36*$^{-/-}$ mice (c, n=6, 6, 5, 5 from left to right group; d, n=6, 6, 4, 5 from left to right group). The livers were collected after 1 hour and hepatic expression of the indicated genes was measured by RT-PCR. **e,** CD36 expression in different cell clusters by analyzing the single-cell sequencing data of human peripheral blood mononuclear cells (PBMCs). **f,** CD36 expression in mouse spleen cells was analyzed by flow cytometry (n=3). **g,** CD36 expression in indicated cell fractions isolated from metastatic liver tumors was analyzed by flow cytometry (n=6). Values for n represent biologically independent samples. Data are mean ± SEM and P values were determined by one-way ANOVA with Tukey's multiple comparison tests (b-d, f) or unpaired two-tailed Student's t-test (g). Source data are provided as a Source Data file.

Supplementary Fig. 3

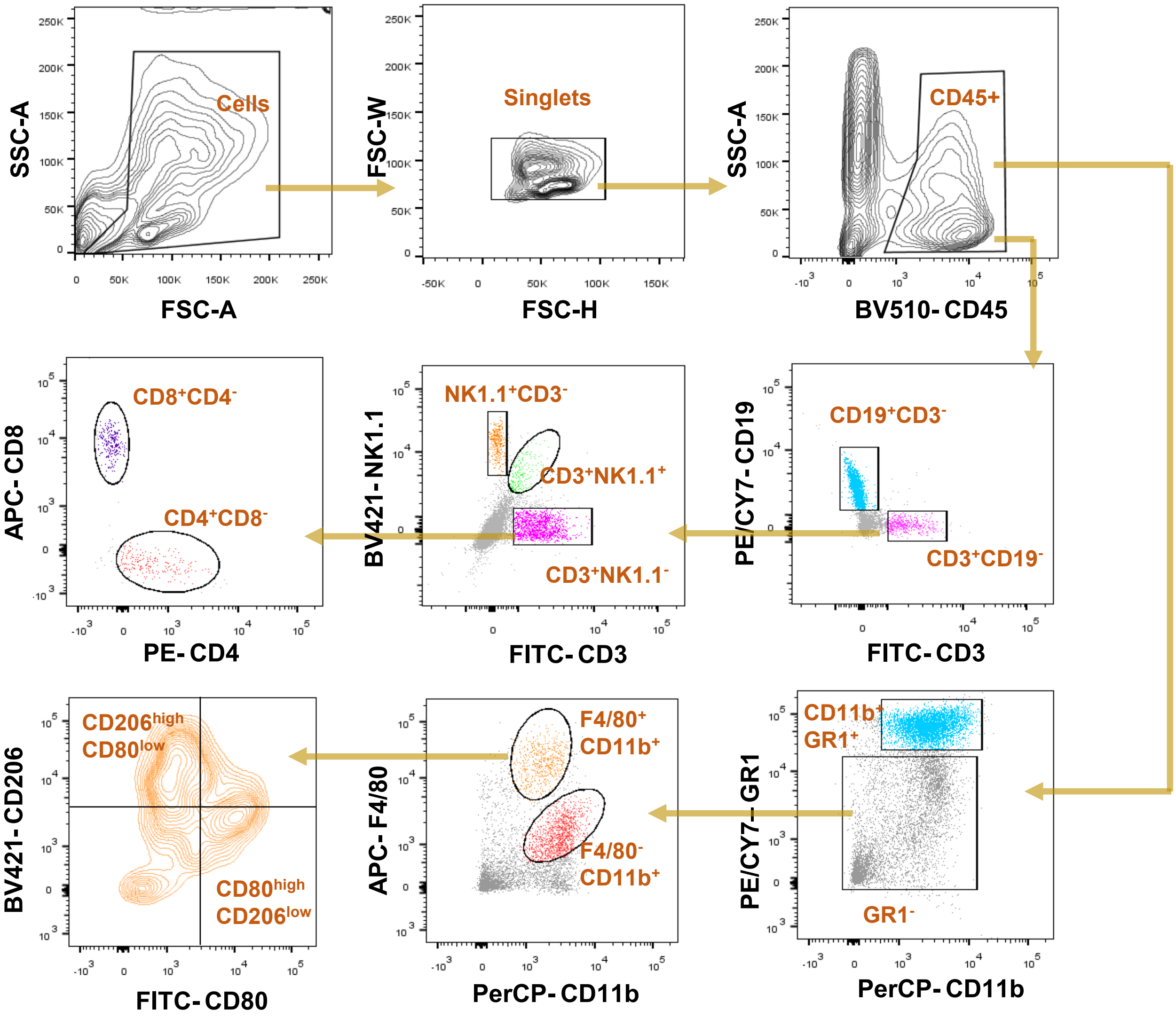

Supplementary Fig. 3 Flow cytometry analysis of tissue infiltrating immune cells. Representative flow cytometry plot showing gating strategy used to analysis B cells ($CD45^{+}CD3^{-}CD19^{+}$), T cells ($CD45^{+}CD3^{+}CD19^{-}NK1.1^{-}$), NK cells ($CD45^{+}CD3^{-}NK1.1^{+}CD19^{-}$), NKT cells ($CD45^{+}CD3^{+}NK1.1^{+}CD19^{-}$), $CD8^{+}$ T cells ($CD45^{+}CD3^{+}CD19^{-}NK1.1^{-}CD4^{-}CD8^{+}$), macrophages or MAMs ($CD45^{+}GR1^{-}F4/80^{+}CD11b^{+}$), inflammatory monocytes (IM, $CD45^{+}GR1^{-}F4/80^{-}CD11b^{+}$), neutrophil or MDSCs ($CD45^{+}CD11b^{+}GR1^{+}$), M1-type MAMs ($CD206^{low}CD80^{high}$), M2-type MAMs ($CD206^{high}CD80^{low}$).

Supplementary Fig. 4

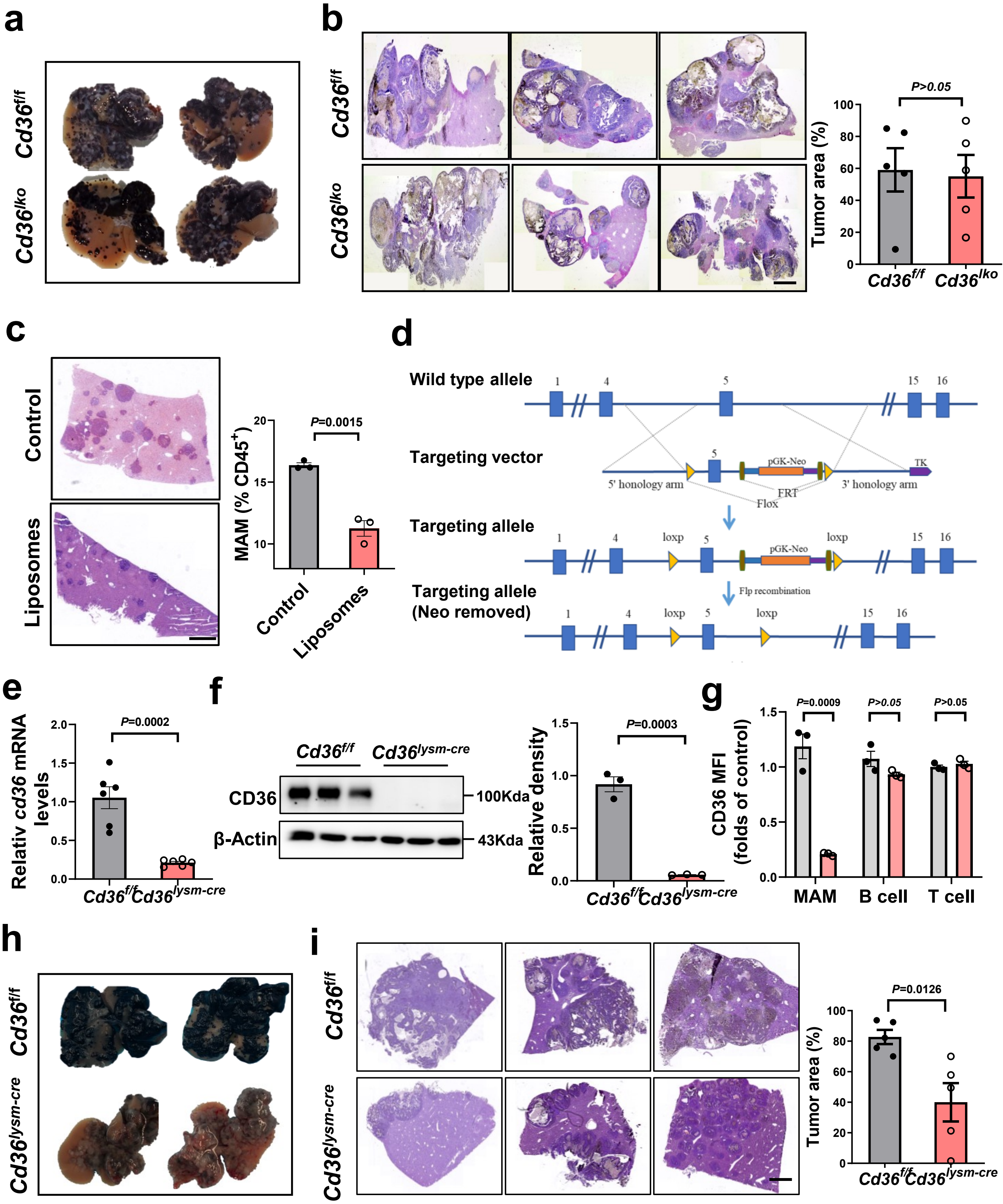

Supplementary Fig. 4 Macrophage CD36 contributes to liver metastasis.

a, Macroscopic images of the liver from *Cd36*^{lko} mice and their littermates after intrasplenic injection of B16F10 cells (n=5). **b,** HE-stained liver sections from *Cd36*^{lko} mice and their littermates. Tumor areas were quantified on the right (n=5). **c,** Tumor appearance (left) and MAM infiltration (right) in mice treated with clodronate liposomes (n=3)**. d,** Schematic of the targeting strategy used to generate CD36$^{f/f}$ mice. **e, f,** The mRNA (n=6) and protein levels (n=3) of CD36 in the BMDMs isolated from CD36$^{f/f}$ and *Cd36*$^{lysm\text{-}cre}$ mice. **g,** The MFI of CD36 in indicated cell populations isolated from metastatic liver tumors (n=3). **h,** Macroscopic images of the liver from *Cd36*$^{lysm\text{-}cre}$ mice and their littermates after intrasplenic injection of B16F10 cells (n=5). **i,** HE staining in the liver sections from *Cd36*$^{lysm\text{-}cre}$ mice and their littermates along with the quantification (n=5). Data are representative of two independent experiments with similar results (a, b, h, i). Values for n represent biologically independent samples. Scale bar, 1000 µm. Data are mean ± SEM and P values were determined by unpaired two-tailed Student's t-test. Source data are provided as a Source Data file.

Supplementary Fig. 5

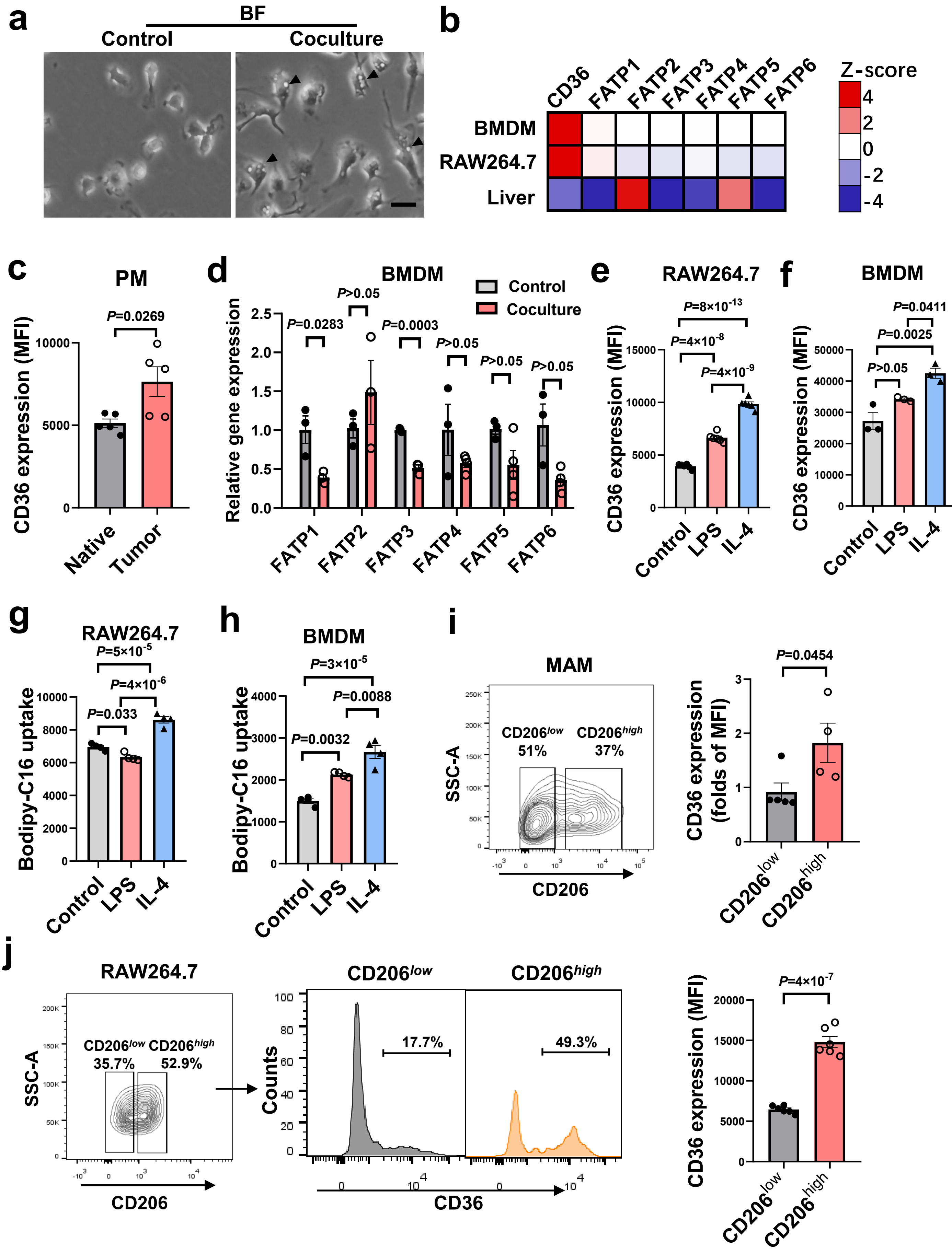

Supplementary Fig. 5 CD36 mediates fatty acid uptake in M2-type macrophage.

a, Typical lipid load in BMDMs cocultured with LLC cells under bright field (BF, n=5). Scale bar, 25 μm. **b,** Comparison of the expression of fatty acid transports in BMDMs, RAW264.7 cells and mouse livers using BioGPS datasets. **c,** The MFI of CD36 in peritoneal macrophages (PM) isolated from mice with or without liver metastasis (n=5). **d,** The expression of FATP1-6 in BMDMs cocultured with or without LLC cells (n=3). **e, f,** The MFI of CD36 in RAW264.7 cells or BMDMs treated with LPS or IL-4 (e, n=6; f, n=3). **g, h,** Fatty acid uptake in RAW264.7 cells or BMDMs (n=4). i, Gating strategy and quantitative results of the MFI of CD36 staining in the subsets of MAMs (n=6). j, Gating strategy, representative histogram and quantitative results of the MFI of CD36 staining in the subsets of RAW264.7 cells (n=5). Data are representative of two independent experiments with similar results (a, e, f, j). Values for n represent biologically independent samples. Data are mean ± SEM and P values were determined by one-way ANOVA with Tukey's multiple comparison tests (e-h) or unpaired two-tailed Student's t-test (c, d, i, j). Source data are provided as a Source Data file.

Supplementary Fig. 6

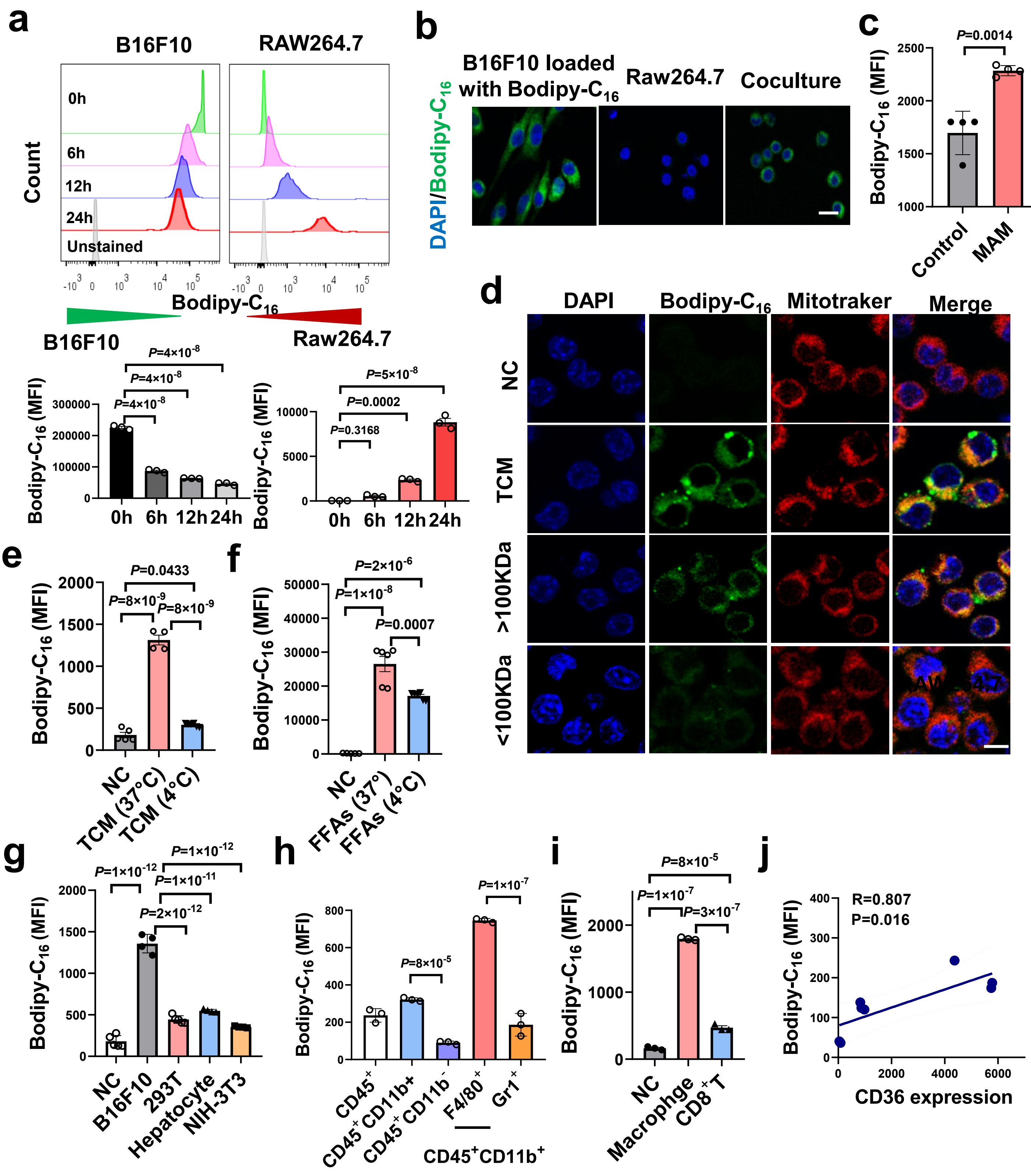

Supplementary Fig. 6 Tumor cell-derived lipids are partitioning into macrophages.

a, RAW264.7 cells were cocultured with B16F10 cell that were preloaded with Bodipy-C_{16} (1μM) in a Transwell chamber. The MFI of Bodipy in these cells was determined by flow cytometry at indicated times (n=3). **b,** Representative images showing the lipid transfer from Bodipy-C_{16}-labled B16F10 tumor cells to RAW264.7 cells (n=5). Scale bar, 20 μm. **c,** The MFI of Bodipy in hepatic macrophages after intrasplenically injected with Bodipy-C_{16}-labled LLC cells (1×10^{6}) for 24h (n=4). **d,** Localization of the incorporated lipids with MitoTracker red in RAW264.7 cells after incubation with different fractions (>100 Kda and <100 Kda) of TCM (n=5). Scale bar, 10 μm. **e, f,** The effects of temperature on TCM lipids (e, n=5, 4, 6 from left to right group) or free fatty acids (f, n=5, 6, 6 from left to right group) uptake by RAW264.7 cells. **g,** The ability of different cell types to transfer their lipids to RAW264.7 cells (n=5, 5, 4, 4, 4 from left to right group). **h,** The intensity of Bodipy in indicated cell fractions after incubation with TCM for 2 h (n=3). **i,** The intensity of Bodipy in purified macrophages and $CD8^{+}T$ after incubation with TCM for 2 h (n=3). **j,** The correlation of CD36 expression with Bodipy intensity in isolated immune cells (n=8). P value was calculated by Pearson correlation analysis. Data are representative of two independent experiments with similar results (a, b, e-h). Values for n represent biologically independent samples. Data are mean ± SEM and P values were determined by one-way ANOVA with Dunnett's (a) or with Tukey's multiple comparison tests (e-g, i, k) or unpaired two-tailed Student's t-test (c, h). Source data are provided as a Source Data file.

Supplementary Fig. 7

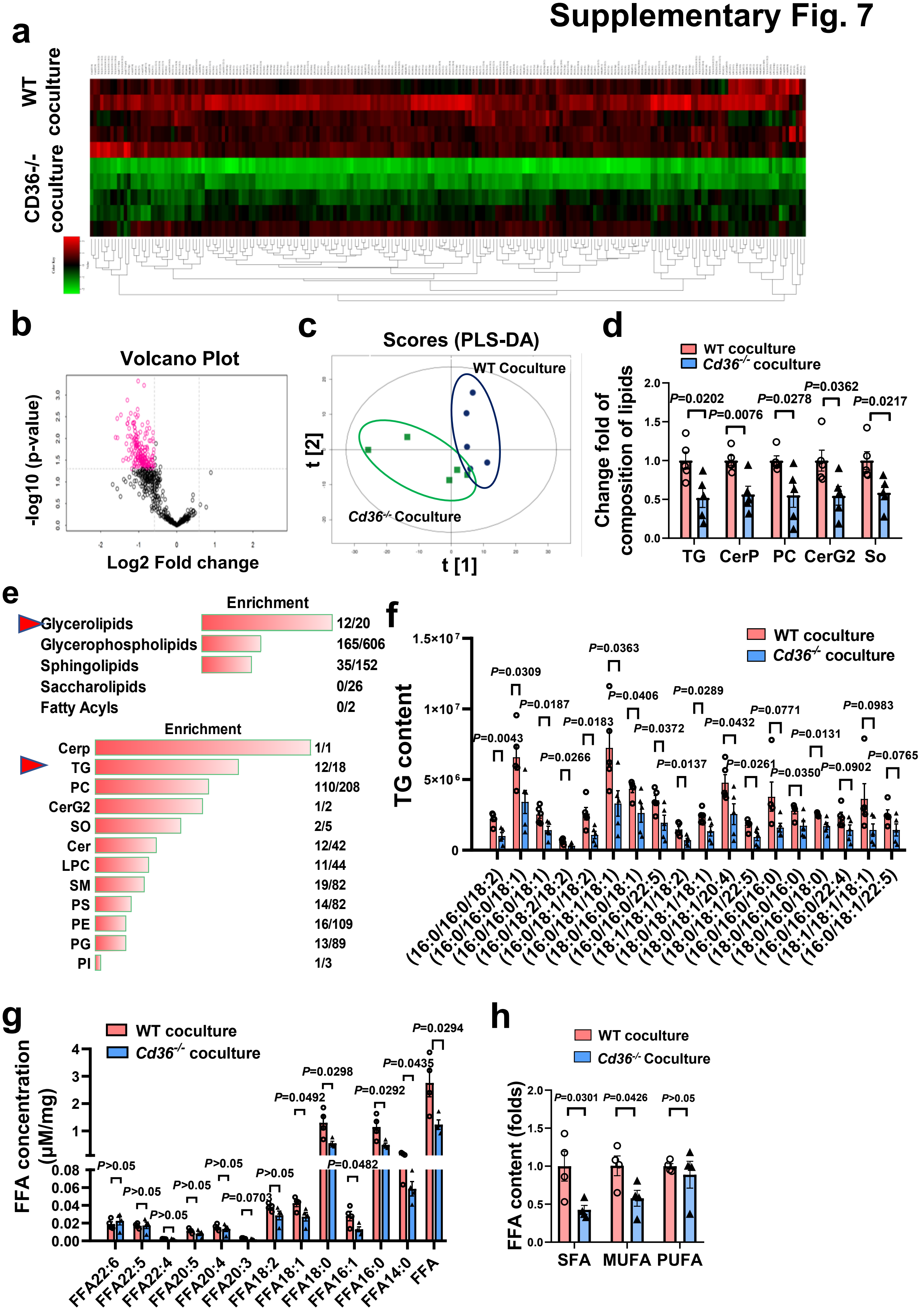

Supplementary Fig. 7 Lipidome profiles is disturbed significantly by CD36.

Lipidome profiling was performed on WT and *Cd36*$^{-/-}$ BMDMs cocultured with LLC cells (n=5). **a,** Heat map analysis for identified lipid species. **b,** The volcano plot showed significantly changed lipids with $P<0.05$, VIP>1 and FC<0.67. P values were determined by unpaired two-tailed Student's t-test. **c,** The PLS-DA score plots differentiated WT and *Cd36*$^{-/-}$ BMDMs. **d,** Different lipid classes between WT and *Cd36*$^{-/-}$ BMDMs. **e,** Enriched different lipid classes between WT and *Cd36*$^{-/-}$ BMDMs. **f,** TG concentrations of WT and *Cd36*$^{-/-}$ BMDMs. **g,** Free fatty acid concentrations of WT and *Cd36*$^{-/-}$ BMDMs (n=4). **h,** Fatty acid saturation of WT and *Cd36*$^{-/-}$ BMDMs (SFA, saturated fatty acid; MUFA, monounsaturated fatty acid; PUFA, polyunsaturated fatty acid). Values for n represent biologically independent samples. Data are mean ± SEM and P values were determined by unpaired two-tailed Student's t-test (d, f, g, h). Source data are provided as a Source Data file.

Supplementary Fig. 8

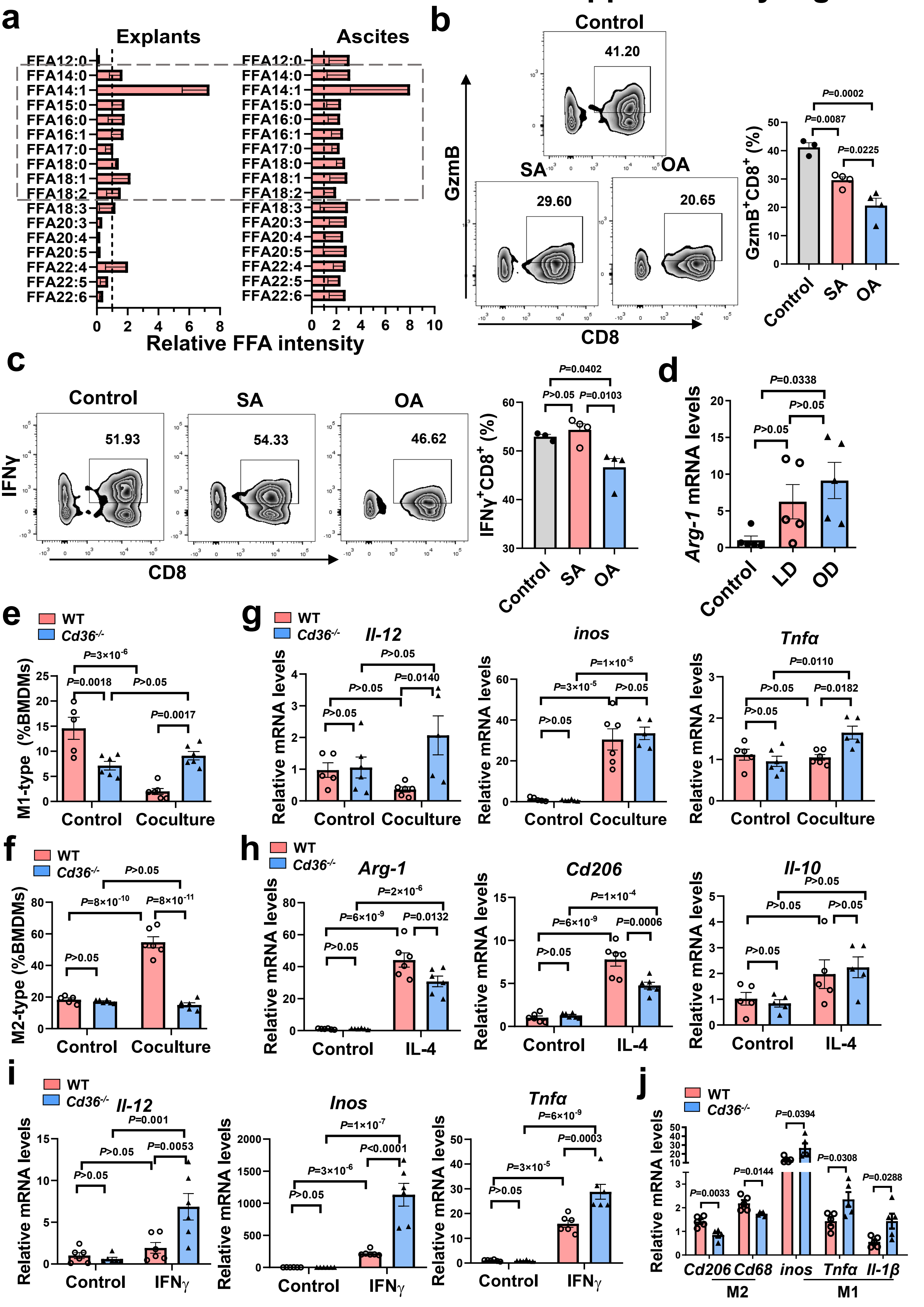

Supplementary Fig. 8 Effects of CD36 on M1/M2 switch. a, Free fatty acid analysis of the CM from metastatic tumor explants compared with normal liver explants, and ascites from liver metastasis bearing mice compared with normal mice (n=2, 3, respectively). **b, c,** After BMDMs were treated with SA or OA, $CD8^+$ T cells were cocultured with BMDMs (10:1) for 72 hours, and the production of GzmB **(b)** and IFN-γ **(c)** in $CD8^+$ T cells were assessed (n=3, 4, 4 from left to right group). **d,** The mRNA levels of *Arg-1* in metastatic tumors from the OD- or LD-fed mice (n=5). **e, f,** Percentages of M1-type ($CD206^{low}CD80^{high}$) or M2-type ($CD206^{high}CD80^{low}$) BMDMs cocultured with or without LLC cells (n=5, 6, 6, 6 from left to right group). **g,** The mRNA levels of indicated cytokines in WT and *Cd36*$^{-/-}$ BMDMs cocultured with or without LLC cells (n=5, 6, 6, 5 from left to right group). **h, i,** The mRNA levels of indicated cytokines in WT and *Cd36*$^{-/-}$ BMDMs treated with IL-4 or IFNγ (h, n=6, 6, 5 for *Arg-1, Cd206, Il-10* respectively; i, n=6). **j,** The mRNA levels of indicated cytokines in metastatic tumors from the WT and *Cd36*$^{-/-}$ mice (n=5). Data are representative of two independent experiments with similar results (g-j). Values for n represent biologically independent samples. Data are mean ± SEM and P values were determined by one-way ANOVA with Tukey's multiple comparison tests (b-f) or unpaired two-tailed Student's t-test (g, h). Source data are provided as a Source Data file.

Supplementary Fig. 9

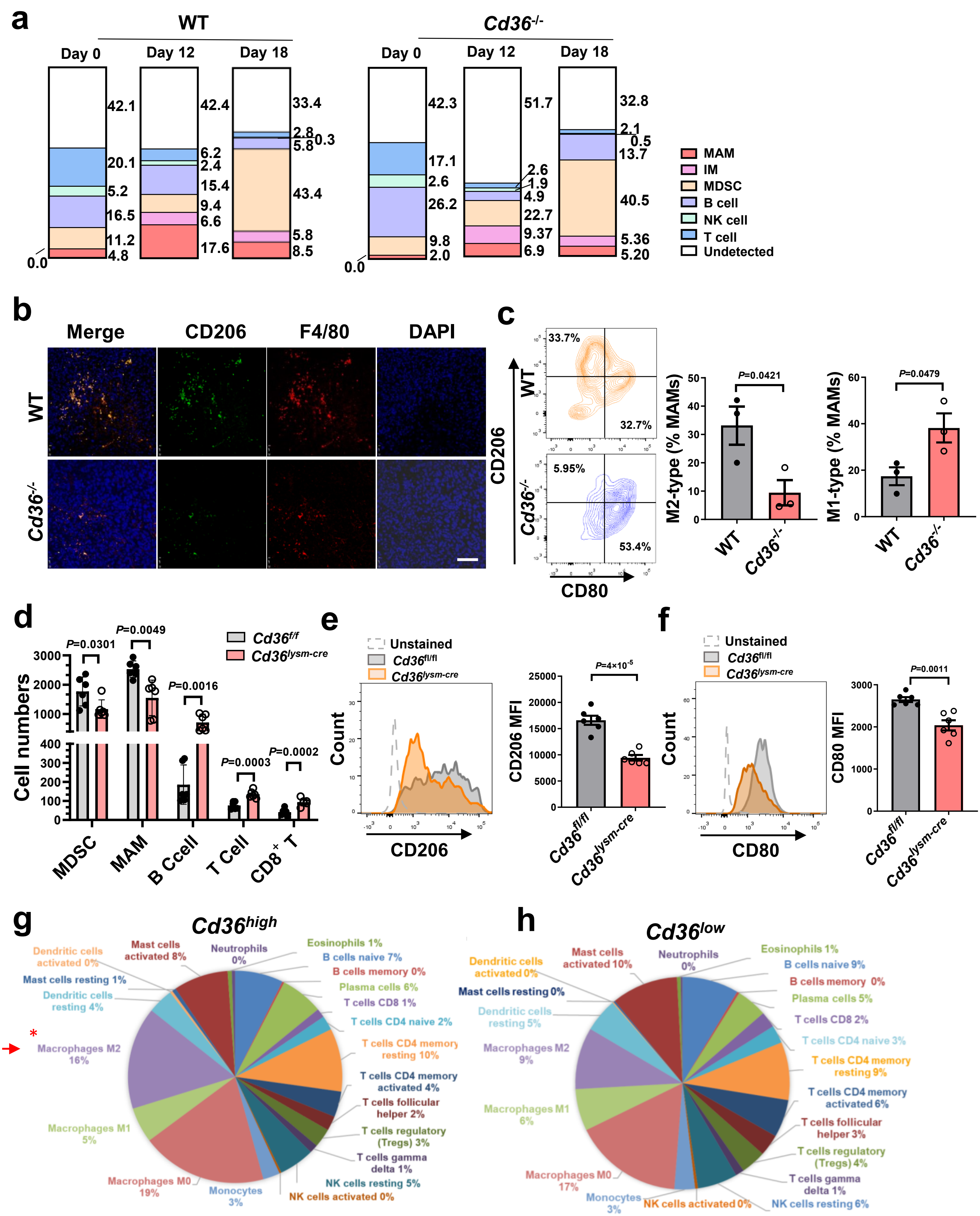

Supplementary Fig. 9 Effects of CD36 on TME.

a, Distribution of immune cells in normal livers or metastatic liver tumors of WT and *Cd36*$^{-/-}$ mice (n=3). **b,** Immunofluorescence images showing F4/80^{+}/CD206^{+} double positive macrophage in metastasized foci of WT and *Cd36*$^{-/-}$ mice (n=5). Scale bar, 200 μm. **c,** Representative plots and percentages of the indicated cells among total MAMs from WT and *Cd36*$^{lysm\text{-}cre}$ mice (n=3). **d,** Numbers of indicated cell populations in the metastatic liver tumors of WT and *Cd36*$^{lysm\text{-}cre}$ mice on day 18 (n=6). **e, f,** Representative histogram (left) and quantitative results of the MFI of CD206 or CD80 staining in MAMs (n = 6). **g, h,** Immune cell infiltration landscapes in liver metastasis patients with colon carcinoma (GSE68468) were analyzed by CIBERSORT between *Cd36*^{low} (n=18) and CD36^{high} (n=18) expression groups. Values for n represent biologically independent samples. Data are mean ± SEM and P values were determined by one-way ANOVA with unpaired two-tailed Student's t-test (c-f). Source data are provided as a Source Data file.

Supplementary Fig. 10

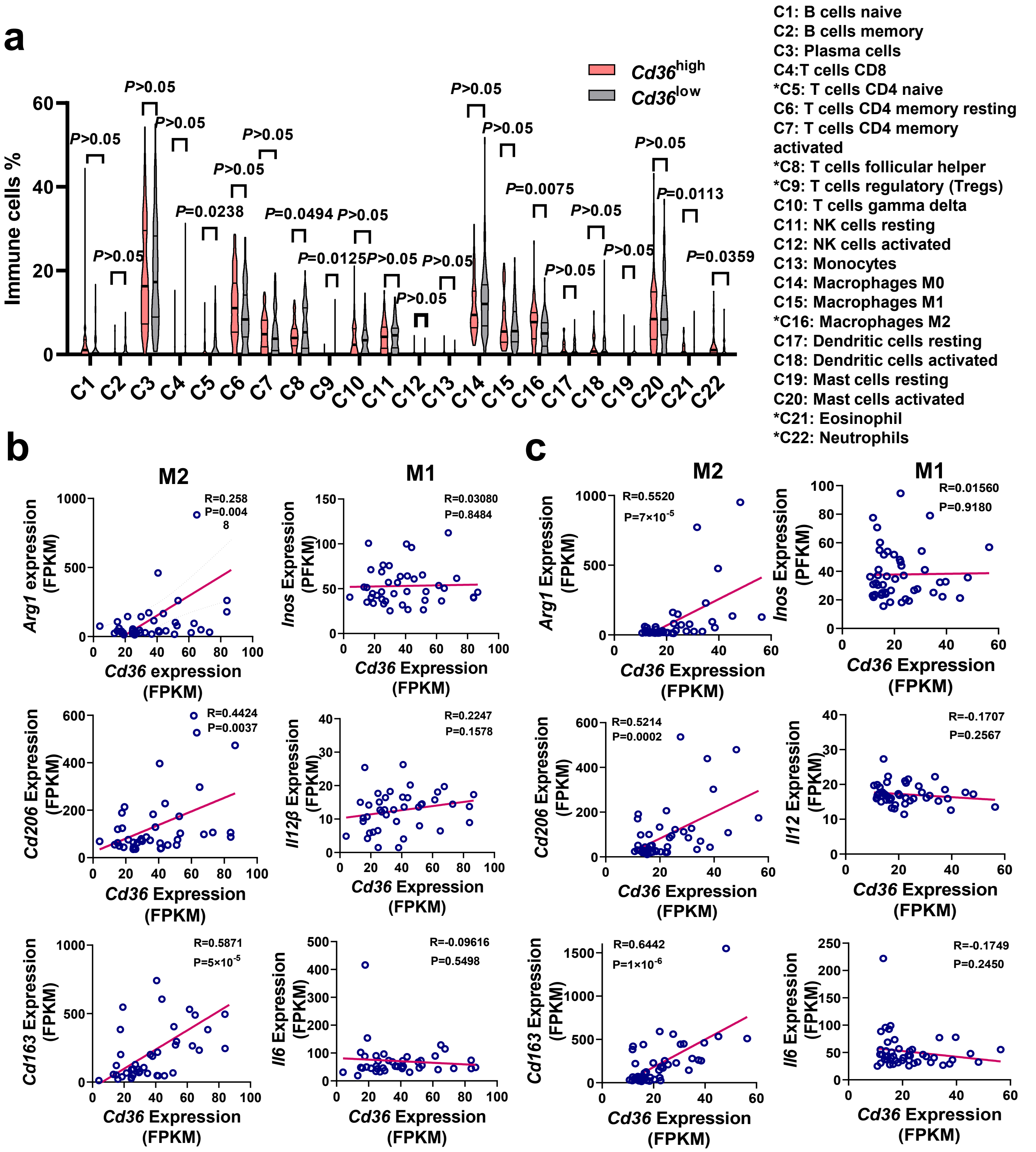

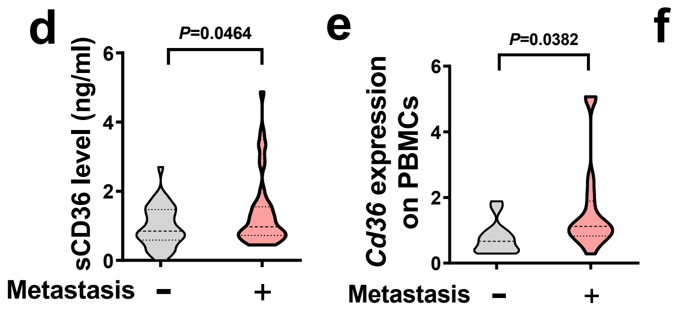

f

The correlation of PBMC *Cd36* with liver cancer metastasis

Metastasis	*Cd36* expression		P value
	High (n=16)	Low (n=16)	
**Absent**	4	10	0.033
**Present**	12	6	

Supplementary Fig. 10 The correlation of CD36 with macrophage polarization in patients with liver metastasis. a, Immune cell infiltration landscapes in liver metastasis patients with colon carcinoma (GSE14095) were analyzed by CIBERSORT between *Cd36*^{low} (n=58) and CD36^{high} (n=58) expression groups. P values were determined by unpaired two-tailed Student's t-test. **b, c,** The correlation of CD36 with M1 or M2 macrophage markers in patients with liver metastasis from GSE68468 (n=41) and GSE41258 (n=46). P value was calculated by Pearson correlation analysis. **d,** Plasma circulating CD36 (sCD36) levels in liver cancer patients with (n=40) or without (n=35) intrahepatic metastasis. Each symbol represents one individual. Data are mean ± SEM and P values were determined by unpaired two-tailed Student's t-test. **e,** The mRNA expression of *Cd36* on PBMCs in liver cancer patients with (n=17) or without (n=14) metastasis. Each symbol represents one individual. Data are mean ± SEM and P values were determined by unpaired two-tailed Student's t-test. **f,** The correlation of PBMC *Cd36* with liver metastasis. P value was calculated by Chi-square analysis. Source data are provided as a Source Data file.

Supplementary Table 1 Primers used for PCR.

Gene	**Species**	**Sequence**
Cd36	Mouse	Forward: 5'-ATGGGCTGTGATCGGAACTG-3'
		Reverse: 5'-TTTGCCACGTCATCTGGGTTT-3'
Il-10	Mouse	Forward: 5'-GCTCTTACTGACTGGCATGAG3'
		Reverse: 5'-CGCAGCTCTAGGAGCATGTG3'
Inos	Mouse	Forward: 5'-GGAGTGACGGCAAACATGACT-3'
		Reverse: 5'-TCGATGCACAACTGGGTGAAC-3'
Arg1	Mouse	Forward: 5'-TGTGAAGAACCCACGGTCTG-3'
		Reverse: 5'-CCAGCACCACACTGACTCTT-3'
Tnfα	Mouse	Forward: 5‘-CCCTCACACTCAGATCATCTTCT-3’
		Reverse: 5‘-GCTACGACGTGGGCTACAG-3’
Il-1β	Mouse	Forward: 5‘-GCAACTGTTCCTGAACTCAACT-3’
		Reverse:5‘- ATCTTTTGGGGTCCGTCAACT -3’
Cd206	Mouse	Forward: 5'-GAGGGAAGCGAGAGATTATGGA-3'
		Reverse: 5'-GCCTGATGCCAGGTTAAAGCA-3'
Il-12	Mouse	Forward: 5'-TGGTTTGCCATCGTTTTGCTG-3’
		Reverse: 5'-ACAGGTGAGGTTCACTGTTTCT-3’
Il-6	Mouse	Forward: 5'-TAGTCCTTCCTACCCCAATTTCC-3'
		Reverse: 5'-TTGGTCCTTAGCCACTCCTTC-3'
E-selectin	Mouse	Forward: 5'-CTCGGGCATGTGGAATGAC-3'
		Reverse: 5'-TGCATTGGTACACGAAGCTGT-3'
Icam-1	Mouse	Forward: 5'-AACAGAATGGTAGACAGCA-3'
		Reverse: 5'-TCCACCGAGTCCTCTTAG-3'
Vcam-1	Mouse	Forward: 5'-GAATGAGGGGGCCAAATCCA-3'
		Reverse: 5'-GACAGGTCTCCCATGCACAA-3'
Fatp1	Mouse	Forward: 5'-CGCTTTCTGCGTATCGTCTG-3'
		Reverse: 5'-GATGCACGGGATCGTGTCT-3'
Fatp2	Mouse	Forward: 5'-TCCTCCAAGATGTGCGGTACT-3'
		Reverse: 5'-TAGGTGAGCGTCTCGTCTCG-3'
Fatp3	Mouse	Forward: 5'- GGCCCGGATTTCCTTTGGATT-3'
		Reverse: 5'-CCCATAGGTGGAGCCCCAT-3'
Fatp4	Mouse	Forward: 5'-ACTGTTCTCCAAGCTAGTGCT-3'
		Reverse: 5'-GATGAAGACCCGGATGAAACG-3'
Fatp5	Mouse	Forward: 5'-CTACGCTGGCTGCATATAGATG-3'
		Reverse: 5'-CCACAAAGGTCTCTGGAGGAT-3'
Fatp6	Mouse	Forward: 5'-CTCCAACCTTCGCTTCGATTC-3'
		Reverse: 5'-TCTGACGTGTTTTGGGAGACT-3'
Cd68	Mouse	Forward: 5'-TGTCTGATCTTGCTAGGACCG-3'
		Reverse: 5'- GAGAGTAACGGCCTTTTTGTGA- 3'
Cd36	Human	Forward: 5’-CTTTGGCTTAATGAGACTGGGAC-3'
		Reverse: 5'-GCAACAAACATCACCACACCA-3'
