# Supplementary material for: CD36-mediated metabolic crosstalk between tumor cells and macrophages affects liver metastasis
Source: Nat Commun. 2022 Oct 2;13:5782. doi: 10.1038/s41467-022-33349-y (PMC9527239; doi:10.1038/s41467-022-33349-y)
Supplement: Supplementary file 2 — Reporting Summary [file 41467_2022_33349_MOESM2_ESM.pdf]

nature portfolio

Corresponding author(s): Xiong Z Ruan

Last updated by author(s): Aug 31, 2022

Reporting Summary

Nature Portfolio wishes to improve the reproducibility of the work that we publish. This form provides structure for consistency and transparency in reporting. For further information on Nature Portfolio policies, see our Editorial Policies and the Editorial Policy Checklist.

Statistics

For all statistical analyses, confirm that the following items are present in the figure legend, table legend, main text, or Methods section.

n/a	Confirmed	
☐	☒	The exact sample size (*n*) for each experimental group/condition, given as a discrete number and unit of measurement
☐	☒	A statement on whether measurements were taken from distinct samples or whether the same sample was measured repeatedly
☐	☒	The statistical test(s) used AND whether they are one- or two-sided<br>*Only common tests should be described solely by name; describe more complex techniques in the Methods section.*
☒	☐	A description of all covariates tested
☐	☒	A description of any assumptions or corrections, such as tests of normality and adjustment for multiple comparisons
☐	☒	A full description of the statistical parameters including central tendency (e.g. means) or other basic estimates (e.g. regression coefficient) AND variation (e.g. standard deviation) or associated estimates of uncertainty (e.g. confidence intervals)
☐	☒	For null hypothesis testing, the test statistic (e.g. *F*, *t*, *r*) with confidence intervals, effect sizes, degrees of freedom and *P* value noted<br>*Give P values as exact values whenever suitable.*
☒	☐	For Bayesian analysis, information on the choice of priors and Markov chain Monte Carlo settings
☒	☐	For hierarchical and complex designs, identification of the appropriate level for tests and full reporting of outcomes
☐	☒	Estimates of effect sizes (e.g. Cohen's *d*, Pearson's *r*), indicating how they were calculated

Our web collection on statistics for biologists contains articles on many of the points above.

Software and code

Policy information about availability of computer code

Data collection	Flow cytometry: FACSAria II (BD Biosciences); Confocal microscopy: Leica TCS SP8 Laser Scanning Confocal Microscope; RT-PCR: CFX Connect Real-time System (BIO-RAD), Q-Exactive mass spectrometer (Thermo ScientificTM).
Data analysis	The following software was used in this study: FlowJo V10, GraphPad Prism 8, Image J 1.47, LipidSearch software 4.2, SIMPCA-P 16.1.

For manuscripts utilizing custom algorithms or software that are central to the research but not yet described in published literature, software must be made available to editors and reviewers. We strongly encourage code deposition in a community repository (e.g. GitHub). See the Nature Portfolio guidelines for submitting code & software for further information.

Data

Policy information about availability of data

All manuscripts must include a data availability statement. This statement should provide the following information, where applicable:

- Accession codes, unique identifiers, or web links for publicly available datasets
- A description of any restrictions on data availability
- For clinical datasets or third party data, please ensure that the statement adheres to our policy

The single-cell sequencing data are obtained from the Human Protein Atlas 38. Cd68 or Cd36 gene expression in different single cell type clusters of the liver was available from v21.1 proteinatlas.org (https://www.proteinatlas.org/ENSG00000129226-CD68/single+cell+type/liver or https://www.proteinatlas.org/ENSG00000135218-CD36/single+cell+type/liver). Cd14 or Cd36 expression in different single cell type clusters of peripheral blood mononuclear cells (PBMCs) was available from v21.1 proteinatlas.org (https://www.proteinatlas.org/ENSG00000170458-CD14/single+cell+type/PBMC or https://www.proteinatlas.org/ENSG00000135218-CD36/single+cell+type/PBMC). RNA-seq data are accessible at the Gene Expression Omnibus (GEO) under accession numbers: GSE68468, GSE14095, GSE41258. All other relevant data supporting the key findings of this study are available within the article, Supplementary Information, or Source Data

file. Source data are provided with this paper.

Field-specific reporting

Please select the one below that is the best fit for your research. If you are not sure, read the appropriate sections before making your selection.

- [x] Life sciences
- [ ] Behavioural & social sciences
- [ ] Ecological, evolutionary & environmental sciences

For a reference copy of the document with all sections, see nature.com/documents/nr-reporting-summary-flat.pdf

Life sciences study design

All studies must disclose on these points even when the disclosure is negative.

Sample size	No sample size calculation was performed. Sample sizes were chosen to satisfy statistical power based on previous experience and knowledge (PMID: 30033199; PMID: 34083525). At least 5 mice/group was used in this study.
Data exclusions	No data was excluded.
Replication	All replications were successful, and the detailed information was provided in corresponding figure legends.
Randomization	All allocations were random in this study.
Blinding	Not applicable since there was no specific grouping.

Reporting for specific materials, systems and methods

We require information from authors about some types of materials, experimental systems and methods used in many studies. Here, indicate whether each material, system or method listed is relevant to your study. If you are not sure if a list item applies to your research, read the appropriate section before selecting a response.

Materials & experimental systems

n/a	Involved in the study	
	x	Antibodies
	x	Eukaryotic cell lines
x		Palaeontology and archaeology
	x	Animals and other organisms
	x	Human research participants
x		Clinical data
x		Dual use research of concern

Methods

n/a	Involved in the study	
x		ChIP-seq
	x	Flow cytometry
x		MRI-based neuroimaging

Antibodies

Antibodies used	anti-ki67 (#27309-1-AP, proteintech), anti-CD31 (#77699, CST), anti-F4/80 (#ab6640, Abcam), anti-CD206 (#abs125294, absin), anti-CD36 (#NB400-144, Novus Biologicals), anti-CD3 (# 553057, BD Pharmingen), anti-CD28 (#553294, BD Pharmingen), Fc block antibody (#14-0161-82, eBioscience), BV510 anti-mouse CD45 antibody (#103137, Biolegend), PerCP anti-mouse CD11b (#101229, Biolegend), APC anti-mouse F4/80 (#17-4801-80, eBioscience) , PE/Cy7 anti-mouse Gr1 (#108415, Biolegend), FITC anti-mouse CD3 (#11-0032-82, eBioscience), PE/Cy7 anti-mouse CD19 (#25-0193-81, eBioscience), BV421 anti-mouse NK1.1 (#108741, Biolegend), PE anti-mouse CD36 (#562702, BD Biosciences), PE anti-mouse CD4 (#4329629, Invitrogen), APC anti-mouse CD8a (#17-0081-81, eBioscience), BV421 anti-mouse CD206 (#141717, Biolegend), FITC anti-mouse CD80 (#FITC-65076, Proteintech), FITC anti-mouse GzmB (#372206, Biolegend), FE anti-mouse IFNγ (#PE-65153, Proteintech).
Validation	All antibodies were used per commercial sources and chosen based upon available validation studies. Available on the manufacturers' websites:<br>Anti-ki67 (https://www.ptgcn.com/products/KI67-Antibody-27309-1-AP.htm)<br>Anti-CD31(https://www.cellsignal.cn/products/primary-antibodies/cd31-pecam-1-d8v9e-xp-rabbit-mab/77699?site-search-type=Products&N=4294956287&Ntt=77699&fromPage=plp&_requestid=512516)<br>Anti-F4/80(https://www.abcam.cn/f480-antibody-cia3-1-macrophage-marker-ab6640.html)<br>Anti-CD206(https://www.absin.cn/rabbit-anti-mrc1-polyclonal-antibody/abs125294.html)<br>Anti-CD36(https://www.novusbio.com/products/cd36-antibody_nb400-144)<br>Anti-CD3(https://www.bdbiosciences.com/en-us/products/reagents/flow-cytometry-reagents/research-reagents/single-color-antibodies-ruo/purified-na-le-hamster-anti-mouse-cd3e.553057)<br>Anti-CD28(https://www.bdbiosciences.com/en-us/products/reagents/flow-cytometry-reagents/research-reagents/single-color-antibodies-ruo/purified-na-le-hamster-anti-mouse-cd28.553294)<br>Fc block antibody(https://www.thermofisher.cn/cn/zh/antibody/product/CD16-CD32-Antibody-clone-93-Monoclonal/14-0161-82)

BV510 anti-mouse CD45 antibody (https://www.biolegend.com/en-us/products/brilliant-violet-510-anti-mouse-cd45-antibody-7995)
PerCP anti-mouse CD11b(https://www.biolegend.com/en-us/products/percp-anti-mouse-human-cd11b-antibody-4315)
APC anti-mouse F4/80(https://www.thermofisher.cn/cn/zh/antibody/product/F4-80-Antibody-clone-BM8-Monoclonal/17-4801-80)
PE/Cy7 anti-mouse Gr1(https://www.biolegend.com/en-us/products/pe-cyanine7-anti-mouse-ly-6g-ly-6c-gr-1-antibody-1931)
FITC anti-mouse CD3(https://www.thermofisher.cn/cn/zh/antibody/product/CD3-Antibody-clone-17A2-Monoclonal/11-0032-82)
PE/Cy7 anti-mouse CD19 （https://www.thermofisher.cn/cn/zh/antibody/product/CD19-Antibody-clone-eBio1D3-1D3-Monoclonal/25-0193-81）
BV421 anti-mouse NK1.1 （https://www.biolegend.com/en-us/products/brilliant-violet-421-anti-mouse-nk-1-1-antibody-7150）
PE anti-mouse CD36 （https://www.bdbiosciences.com/en-us/products/reagents/flow-cytometry-reagents/research-reagents/single-color-antibodies-ruo/pe-mouse-anti-mouse-cd36.562702）
APC anti-mouse CD8a （https://www.thermofisher.cn/cn/zh/antibody/product/CD8a-Antibody-clone-53-6-7-Monoclonal/17-0081-81）
BV421 anti-mouse CD206 （https://www.biolegend.com/en-us/products/brilliant-violet-421-anti-mouse-cd206-mmr-antibody-8638）
FITC anti-mouse CD80 （https://www.ptgcn.com/products/CD80--B7-1--Antibody-FITC-65076.htm）
FITC anti-mouse GzmB （https://www.biolegend.com/en-us/products/fitc-anti-human-mouse-granzyme-b-recombinant-antibody-14430）
FE anti-mouse IFNγ （https://www.ptgcn.com/products/IFN-gamma-Antibody-PE-65153.htm）

Eukaryotic cell lines

Policy information about cell lines

Cell line source(s)	Murine Lewis lung carcinoma line LLC, melanoma cell line B16F10 and hepatoma cell line Hepa1-6 were purchased from Cell Bank of Chinese Academy of Sciences (Shanghai, China). Colon carcinoma cell line CT26 was purchased from ATCC (Rockville, MD, USA).The macrophage cell line RAW264.7 was kindly provided by JH. Yan (Chongqing Medical University, China) that was purchased from Cobioer Biosciences Co., Ltd (Nanjing, China).
Authentication	The cell lines were authenticated using STR method by the suppliers.
Mycoplasma contamination	Mycoplasma testing was negative.
Commonly misidentified lines (See ICLAC register)	No commonly misidentified cell lines were used in the study.

Animals and other organisms

Policy information about studies involving animals; ARRIVE guidelines recommended for reporting animal research

Laboratory animals	CD36 knockout (Cd36-/-) mice strain created on a C57BL/6J background were kindly provided by Dr. Maria Febbraio (Lerner Research Institute). Alb-cre+/- mice and Lysm-cre+/+ mice were obtained from Shanghai Research Center for Model Organisms (China). Cd36f/f mice, in which the exon 5 of the CD36 allele was flanked with loxP recombination sites were generated and crossed with either Alb-cre+/- or Lysm-cre+/+ mice to generate hepatocyte- or myeloid-specific CD36 knockout mice. Mice were maintained in a controlled environment of 20–23°C, with a 12/12 h light/dark cycle, 50–60% humidity, and food and water provided ad libitum. Male, 8-10 weeks old mice as described above were used for this study.
Wild animals	The study did not involve wild animals.
Field-collected samples	The study did not involve samples collected from the field.
Ethics oversight	Animal care and experimental procedures were performed with approval from the animal care committees of Chongqing Medical University.

Note that full information on the approval of the study protocol must also be provided in the manuscript.

Human research participants

Policy information about studies involving human research participants

Population characteristics	Patients who were identified using the Barcelona Clinic Liver Cancer guidelines definition and underwent surgical treatment at the Second Affiliated Hospital of Chongqing Medical University (Chongqing, China) were included in the study. The average age of these patients was 53 years, 80% were men (n=60) and 20% were women (n=15).
Recruitment	A total of 75 blood samples were collected from patients who were identified using the Barcelona Clinic Liver Cancer guidelines definition and underwent surgical treatment at the Second Affiliated Hospital of Chongqing Medical University (Chongqing, China). These participants were recruited randomly, so there was no potential self-selection bias or other biases that may be present and impact results.
Ethics oversight	The study was approved by the Ethics Committee of the Second Affiliated Hospital of Chongqing Medical University.

Note that full information on the approval of the study protocol must also be provided in the manuscript.

Flow Cytometry

Plots

Confirm that:

- [x] The axis labels state the marker and fluorochrome used (e.g. CD4-FITC).
- [x] The axis scales are clearly visible. Include numbers along axes only for bottom left plot of group (a 'group' is an analysis of identical markers).
- [x] All plots are contour plots with outliers or pseudocolor plots.
- [x] A numerical value for number of cells or percentage (with statistics) is provided.

Methodology

Sample preparation	The freshly isolated liver tumor tissues or normal liver tissues were cut into species and digested in Hanks' buffer containing IV collagenase (Sigma) for 30 min at 37℃. The dissociated cells were filtrated with a 70 μm nylon mesh and centrifuged at 50g for 5min to remove hepatocytes. The suspension was centrifuged at 800g for 5min, and then the pellets were resuspended in red blood cell lysis buffer. Cells were recovered by centrifugation at 800 g, and used for further analysis of liver immune cells.<br>Single-cell suspensions from murine spleens were prepared by mechanical disruption in Hanks' buffer. Cells in suspension were centrifuged for 5 min at 400 g. After red blood cell lysis, cells were filtrated through a 70 μm nylon mesh. CD8+ T cells were isolated from spleen cells using magnetic bead sorting according to manufacturer's instructions (#480008, Biolegend). Spleen macrophages were allowed to adhere for 2h at 37°C and the suspension cells were washed extensively with warm medium prior to use.
Instrument	Data was collected using a FACSAria II flow cytometer (BD Biosciences, USA).
Software	Data was analyzed by a FlowJo V10 software.
Cell population abundance	CD45+GR1-F4/80+CD11b+ MAMs were sorted and the purity was routinely >90%.<br>The purity of CD8+ T cells was routinely >90%.
Gating strategy	Gating strategy used to analysis B cells (CD45+CD3-CD19+), T cells (CD45+CD3+CD19-NK1.1-), NK cells (CD45+CD3-NK1.1 +CD19-), NKT cells (CD45+CD3+NK1.1+CD19-), CD8+ T cells (CD45+CD3+CD19-NK1.1-CD4-CD8+), macrophages or MAMs (CD45+GR1-F4/80+CD11b+), inflammatory monocytes (IM, CD45+GR1-F4/80-CD11b+), neutrophil or MDSCs (CD45+CD11b +GR1+), M1-type MAMs (CD206lowCD80high), M2-type MAMs (CD206highCD80low). FACS-based gating strategy is shown in Supplemental figure 3.

- [x] Tick this box to confirm that a figure exemplifying the gating strategy is provided in the Supplementary Information.
